# PAQR5 drives the malignant progression and shapes the immunosuppressive microenvironment of hepatocellular carcinoma by activating the NF-κB signaling

**DOI:** 10.1186/s40364-025-00785-z

**Published:** 2025-05-07

**Authors:** Ruida Yang, Huanhuan Wang, Cong Wu, Yu Shi, Hanqi Li, Xinyue Bao, Yuqian Yang, Shaoshan Han, Xue Yang, Jie Tao, Hao Sun, Shaobo Wu, Liankang Sun

**Affiliations:** 1 Department of Hepatobiliary Surgery, The First Affiliated Hospital of Xi’an Jiaotong University, Xi’an, 710061 PR China; 2 Department of Thoracic Surgery, National Clinical Research Center for Cancer/Cancer Hospital, National Cancer Center, Chinese Academy of Medical Sciences and Peking Union Medical College, Beijing, China; 3 Department of Oncology, The First Affiliated Hospital of Xi’an Jiaotong University, Xi’an, 710061 PR China; 4 Department of Medical Oncology, Xi’an No.3 Hospital, The Affiliated Hospital of Northwest University, Xi’an, 711018 Shaanxi People’s Republic of China; 5 Honghui Hospital, Xi’an Jiaotong University, Xi’an, 710054 Shaanxi China

**Keywords:** PAQR5, Hepatocellular carcinoma, NF-κB, Single cell-RNA seq, Spatial transcriptomic

## Abstract

**Background:**

Progesterone and adipose Q receptor 5 (PAQR5), a membrane receptor characterized by seven transmembrane domains, has been indirectly implicated in pro-carcinogenic activities, though its specific role in hepatocellular carcinoma (HCC) remains to be defined.

**Methods:**

This study aimed to elucidate the molecular mechanisms by which PAQR5 facilitates HCC progression and contributes to the immunosuppressive microenvironment through an integrative approach combining multi-omics analysis and experimental validation. Utilizing data from bulk, single-cell, and spatial transcriptomics cohorts, this study systematically assessed the expression patterns, immune landscape, and functional characteristics of PAQR5 across different levels of resolution in HCC.

**Results:**

PAQR5 expression was significantly upregulated in tumor tissues and correlated with poor clinical outcomes. Enrichment analysis revealed that PAQR5 activated the NF-κB signaling pathway in HCC. Single-cell transcriptomics identified PAQR5 as predominantly localized within malignant cell clusters, with significant association with NF-κB pathway activation. Spatial transcriptomics further corroborated the alignment of PAQR5 expression with tumor cell distribution. In vitro assays showed elevated PAQR5 levels in HCC cell lines, and silencing PAQR5 significantly suppressed cell proliferation, invasion, epithelial-mesenchymal transition (EMT), and prevented the formation of immunosuppressive microenvironment. In vivo studies demonstrated that targeting PAQR5 attenuated tumorigenic potential, disrupted the invasion-metastasis cascade and inhibited the tumor immune escape. Mechanistically, PAQR5 was found to activate NF-κB signaling by inducing ERK phosphorylation, thereby driving proliferation, invasion, EMT, and immune escape in HCC through the pathway.

**Supplementary Information:**

The online version contains supplementary material available at 10.1186/s40364-025-00785-z.

## Significance

The comprehensive multi-omics analysis and experimental findings delineate a potential oncogenic role for PAQR5 in HCC *via* modulation of the NF-κB pathway, highlighting its relevance in promoting malignant cell behavior and establishing an immunosuppressive tumor microenvironment.

**Supplementary Information:**

The online version contains supplementary material available at 10.1186/s40364-025-00785-z.

## Introduction

Hepatocellular carcinoma (HCC), a predominant form of primary liver cancer, ranks as the third leading cause of cancer-related mortality worldwide, presenting a significant global health challenge [1]. The development and progression of HCC are driven by multiple risk factors, including chronic hepatitis B or C infection, excessive alcohol intake, nonalcoholic fatty liver disease, exposure to aflatoxins, and obesity [2, 3]. Approximately 70-90% of HCC cases arise in individuals with chronic liver disease or cirrhosis [4]. Standard therapeutic approaches for HCC vary by stage, encompassing hepatic resection, liver transplantation, and local ablation for early-stage disease; chemoembolization for intermediate stages; and systemic therapies for advanced cases [5]. The emergence of immunotherapy, particularly agents targeting the programmed cell death protein 1 (PD-1) and programmed cell death ligand-1 (PD-L1) pathway, has transformed HCC management, achieving remarkable clinical success [6]. Despite these therapeutic advancements, prognosis remains dismal due to the high rate of metastasis, with more than 70% of patients experiencing tumor recurrence within five years following surgical resection or ablation [7, 8]. The global HCC burden is further exacerbated by delayed diagnosis and the limited efficacy of current treatments, highlighting an urgent need for improved diagnostic, prognostic, and therapeutic strategies [9–11].

The progestin and adiponectin receptor (PAQR) family encompasses a group of membrane protein receptors characterized by seven transmembrane domains, consisting of 11 members in humans (PAQR1-11) [12–14]. PAQR1-4 belong to the adiponectin-related receptor subgroup, while PAQR5-9 constitute the membrane progesterone receptors (mPRs), which include five subtypes: PAQR5 (mPRγ), PAQR6 (mPRδ), PAQR7 (mPRα), PAQR8 (mPRβ), and PAQR9 (mPRε). These receptors mediate rapid progesterone signaling and play roles in cell cycle regulation and tumor progression [14–17]. Extensive research has focused on mPRs, particularly regarding their functions in reproduction and the central nervous system. Recent studies have linked abnormal mPR expression to the pathogenesis of various cancers [15, 18, 19], including prostate, breast, endometrial, ovarian, and bladder malignancies [17, 20–23]. Increasing evidence indicates that elevated PAQR5 expression is associated with tumorigenesis, progression, and poor prognosis across multiple cancers [15]. However, the oncogenic potential and prognostic implications of PAQR5 in hepatocellular carcinoma have yet to be explored.

HCC arises from the accumulation of genetic and epigenetic alterations that disrupt tumor suppressor gene functions, activate oncogenes [24], and ultimately impair key cellular processes. Several signaling pathways, including Ras/Raf/MAPK, PI3K/Akt/mTOR, JAK/STAT, Wnt/β-catenin, Hippo, Notch, and Hedgehog, are implicated in regulating cell proliferation, differentiation, apoptosis, metastasis, and angiogenesis, all of which contribute to HCC pathogenesis [25–30]. Membrane progesterone receptors (mPRs), which are coupled to G proteins, appear to signal primarily *via* a Gi-mediated pathway [21]. Extensive studies have demonstrated that mPRs mediate progesterone’s dual roles in breast cancer, exerting either pro- or anticancer effects. These receptors influence multiple immune cell types, modulate pro-inflammatory factor expression, and play pivotal roles in regulating tumor cell proliferation, metastasis, and apoptosis, with the effects varying across different tumor contexts [31–35]. However, the role of PAQR5 in HCC progression remains largely undefined, limiting the potential to develop new targeted therapies. This study aimed to clarify the diagnostic and prognostic significance of PAQR5 in HCC through a combination of bioinformatics analysis and experimental validation.

An integrated investigation was conducted using bioinformatics tools and experimental approaches to elucidate PAQR5’s role in HCC. PAQR5 expression was systematically analyzed across multiple cohorts, assessing its association with clinicopathological features and the clinical outcomes of patients with HCC. Expression levels were further validated at the spatial transcriptome scale. This study also explored the relationship between PAQR5 expression and the tumor immune microenvironment, utilizing functional enrichment analyses to identify potential mechanistic pathways. The results revealed that PAQR5 is differentially expressed in HCC tissues and may serve as an independent prognostic biomarker. Additionally, PAQR5 was linked to immune infiltration in HCC, suggesting its involvement in disease progression through several signaling pathways. Experimental validation in Hep3B and MHCC-97 cell lines and animal models confirmed that PAQR5 knockdown inhibits proliferation, invasion, and epithelial-mesenchymal transition (EMT). These findings provide a comprehensive insight into PAQR5’s role in HCC and support its potential as a novel diagnostic, prognostic, and immune-related marker in this malignancy.

## Materials and methods

### Dataset collection and preprocessing

The TCGA-LIHC dataset, consisting of 423 samples (50 normal and 373 tumor samples), was used for this study, including RNA sequencing data and clinical information sourced from the TCGA database (https://portal.gdc.cancer.gov/). Patients lacking survival data were excluded from the TCGA-LIHC analysis. Ensembl IDs were converted to gene symbols for subsequent analysis.

In addition to TCGA data, the GSE39791 dataset (comprising 72 tumor and adjacent tissue samples) and the GSE54236 dataset (consisting of 81 tumor and 80 adjacent tissue samples) were retrieved from the GEO database (https://www.ncbi.nlm.nih.gov/geo/).

For single-cell transcriptomic analysis, the GSE166635 dataset was utilized, which contains 25,189 cells from two patients with HCC, obtained from the GEO database (https://www.ncbi.nlm.nih.gov/geo/). The preprocessing included quality control, cell clustering, differential gene expression analysis, cell type annotation, and identification of malignant cells, following the TISCH workflow [36].

Spatial transcriptomics (ST) data were accessed from PMID: 36,708,811 and can be found in Mendeley Data (identifier: skrx2fz79n), containing three HCC samples (HCC1, HCC2, and HCC3), corresponding to samples P9T, P10T, and P3T in the Mendeley repository [37].

All datasets used are publicly accessible from previously published sources.

### Bulk transcriptomic analysis

PAQR5 expression levels were categorized as high or low based on the median value across all cohorts. Differential expression analysis was conducted in the TCGA-LIHC and GSE39791 datasets, with both paired and unpaired comparisons in the TCGA cohort, as well as in a combined analysis of the TCGA and GTEx cohorts [38].

Survival analysis was conducted to compare high and low PAQR5 expression groups in the TCGA cohort, with Kaplan-Meier curves generated using the ‘survminer’ and ‘survival’ R packages to evaluate the association between PAQR5 expression and HCC prognosis. Additionally, the correlation between PAQR5 expression and clinical variables, such as tumor stage, was assessed within the TCGA dataset.

Immune infiltration analysis for bulk transcriptomes was performed using algorithms including ssGSEA, XCELL, and CIBERSORT [39–41], quantifying the relationship between PAQR5 expression and various components of the immune microenvironment, such as immune cell populations, immune-related molecules, and immune response states in the TCGA cohort.

Patients were stratified into four groups (Q1, Q2, Q3, and Q4) based on quartile values of PAQR5 expression, where Q1 represented the top 25% with the highest expression and Q4 represented the bottom 25% with the lowest expression. Following the methodology of Thorsson V et al. on immune response and genomic characteristics, the average score for each group was calculated while excluding missing values, and the results were visualized using the ‘pheatmap’ package [42].

Enrichment analysis incorporated both KEGG and Gene Set Enrichment Analysis (GSEA) [43, 44]. The TCGA-LIHC cohort was dichotomized into high and low expression groups based on the median PAQR5 expression value. Differential gene expression analysis was carried out using the ‘limma’ package, applying thresholds of log fold change (logFC) > 1.5 and *P* < 0.05 to identify significant differentially expressed genes [45]. These genes were then subjected to KEGG and GSEA enrichment analyses.

To investigate functional states, the CancerSEA database was utilized to classify various functional states across 14 tumor cell types [46]. The activity of specific pathways was assessed by integrating the expression levels of characteristic genes. Using the R package ‘GSVA’ with the z-score parameter, the functional state scores for 14 gene sets were calculated, resulting in a combined z-score to reflect pathway activity. The resulting scores were standardized as gene set scores, and Pearson correlation coefficients were computed between individual gene expression and each functional state gene set score.

### Single-cell RNA sequencing (scRNA-seq) data processing and analysis

Single-cell data processing was performed using the R package “Seurat“ [47]. Cells with gene expression count between 500 and 8000 were retained, while those exhibiting mitochondrial gene expression above 15% were excluded. Gene expression normalization was carried out using the SCTransform method, followed by dimensionality reduction through principal component analysis (PCA). To address inter-sample batch effects, the “Harmony” package was utilized. Clustering analyses were conducted with Seurat functions (FindNeighbors and FindClusters) [48], and results were visualized using Uniform Manifold Approximation and Projection (UMAP). For cell type identification, cell marker gene information specific to liver tissue was obtained from the CellMarker database (http://xteam.xbio.top/CellMarker/index.jsp), improving the accuracy of cell type assignments [49].

The UMAP approach was used to visualize the expression patterns of PAQR5, RELA (p65), and MAPK1 (ERK). The Kruskal-Wallis rank sum test evaluated expression differences of these genes across various cell types.

To examine PAQR5 distribution, cells were categorized as PAQR5-positive or PAQR5-negative based on their expression levels, and the proportion of each cell type within these groups was calculated. Enrichment analysis and cell communication analysis were performed to explore functional pathway differences between the PAQR5-positive and PAQR5-negative subpopulations. The AUCell package assessed pathway activity scores related to immune response, metabolism, signaling, proliferation, and cell death. Cells were grouped as PAQR5-positive or PAQR5-negative for differential score analysis using the limma package. Cells with zero counts in either group were excluded from the analysis. The CellChat package (version 1.6.1) was employed to analyze intercellular communication networks by integrating gene expression data with known ligand-receptor-cofactor interactions [50]. Malignant cells were further divided based on PAQR5 expression, and cell communication analysis was performed to compare signaling differences between these subgroups.

Subsequently, the malignant cell subgroup was analyzed for co-activation of key molecules in the NF-κB pathway, specifically ERK and p65, alongside PAQR5 expression. Pearson correlation coefficients were calculated to assess the relationship between PAQR5 expression and the activation of these key molecules(ERK and p65).

### Spatial transcriptomic analysis

Spatial transcriptome analysis offers insights into the spatial distribution and functional role of PAQR5 within the tumor microenvironment (TME) [51].

To accurately determine the cellular composition of each spot on the 10x Visium slides, the ‘SPOTlight’ package was utilized for deconvolution analysis. Stringent quality control measures were applied to single-cell transcriptome data, filtering based on the number of expressed genes, the unique molecular identifier (UMI) counts, and the percentage of mitochondrial RNA in each cell to ensure data reliability.

The average expression of the top 25 specifically expressed genes was calculated for each cell type in the scRNA-seq reference, creating a signature score matrix for each locus. An enrichment scoring matrix was then generated using the get_enrichment_matrix and enrichment_analysis functions from the ‘Cottrazm’ package, providing robust support for downstream cellular composition analysis. The SpatialFeaturePlot function in Seurat was used to visualize cell type enrichment scores, with darker colors indicating higher enrichment and greater cell type abundance at each spot.

Microregions were classified into three groups: malignant (score of 1 for malignant cells), normal (score of 0), and mixed (all other scores). Statistical differences in gene expression among these subgroups were assessed pairwise using Wilcoxon rank sum tests.

Based on the deconvolution results, the cell type with the highest abundance in each microregion was identified, and the SpatialDimPlot function in Seurat was employed to visualize the most prevalent cellular component in each microregion. The SpatialFeaturePlot function was used to map the expression of PAQR5 across the microregions. Spearman correlation analysis was conducted to assess the relationships between cell type abundance and other cellular components, as well as between cell type abundance and PAQR5 expression across all spots. These correlations were visualized using the ‘linkET’ package, facilitating the identification of spatial associations within the tumor microenvironment.

### ATAC-seq and epigenetic analysis

ATAC-seq (Assay for Transposase-Accessible Chromatin with high-throughput sequencing) was performed to investigate the mechanisms underlying PAQR5 dysregulation at the transcription factor level. Peak annotation was performed using the Peak Transcription Start Site function in the ‘ChIPseeker’ package, while the covplot function generated coverage plots showing the distribution of peaks across chromosomes. To evaluate the correlation between transcription factor expression and ATAC peaks, the Spearman correlation coefficient was calculated, considering peaks within ± 3000 base pairs of the promoter region of the target gene. Only correlations that met statistical significance criteria (*P* < 0.01, cor > 0) were retained for further analysis.

The plotAnnoBar and vennpie functions in ‘ChIPseeker’ were used to visualize peak location types, including Promoter, 5’ UTR, 3’ UTR, Exon, Intron, Downstream, and Intergenic regions, providing insights into the genomic context of chromatin accessibility.

Bayesian colocalization analysis assessed the probability of shared causal variation between PAQR5 and HCC using the ‘coloc’ package with default parameters (https://github.com/chr1swallace/coloc). Colocalization data were extracted with the ieugwasr_to_coloc function, and the coloc.abf function performed genetic colocalization analysis to determine if the genetic causal variation associated with eQTL genes was shared. A colocalization threshold of PP.H4.abf > 80% indicated strong evidence of shared genetic causality. Visualization was achieved using the stack_assoc_plot function in the ‘gassocplot2’ package.

### Chemotherapy drug sensitivity analysis and targeted drug screening

Spearman correlation analysis was also applied to correlate gene expression with dose-response metrics (area under the curve [AUC]) from the CTRP and PRISM databases, as well as with the half-maximal inhibitory concentration (IC50) values for antagonists in the GDSC1 and GDSC2 databases.

To identify potential therapeutic agents that could counteract tumor-promoting effects mediated by PAQR5, cMAP analysis was conducted. The analysis used the cMAP_gene_signatures.rdata file, which contains signatures for 1,288 compounds. A gene-associated signature comprising the top 150 most significantly upregulated and downregulated genes was constructed by comparing tumor samples with high versus low PAQR5 expression. This signature was then compared to the cMAP gene signatures using the XSum (eXtreme Sum) feature-matching method to calculate similarity scores across the 1,288 compounds. The methodology followed protocols outlined in previous research [52, 53].

### Cell culture

Human HCC cell lines (Hep3B, Huh7, MHCC-97 H, SNU-449, HCCLM3, SK-Hep-1, PLC-PRF/5, HepG2) and the normal human liver cell line (MIHA) were sourced from the Cell Bank of the Chinese Academy of Sciences (Shanghai, China). The mice HCC cell line Hepa 1–6 were purchased from Wuhan Procella Biotechnology Co., Ltd. All cells were cultured in Dulbecco’s modified Eagle’s medium (DMEM; Gibco, Grand Island, NY, USA) supplemented with 10% fetal bovine serum (FBS; Gibco) and 1% penicillin–streptomycin (Invitrogen, CA, USA).

### Reagents, plasmids, and antibodies

The PAQR5 shRNA plasmid and full-length PAQR5 overexpression plasmid (PLVX3-flag-PAQR5) were obtained from Santa Cruz Biotechnology, Inc. (#sc-106235) and Tsingke Biotech Co. (Beijing, China), respectively. The shRNA#1(5’-GCAAGCATCAAGGTGAGTTTA-3’), shRNA#2(5’-CAGCCTCAGCAACATTATTTA-3’), and shRNA#3 (5’-CACATTCAGCTCTATGTCCAA) for targeting PAQR5 in mouse HCC cell line: Hepa 1–6 were purchased from Xi’an GeneCarer Biotech Co., Ltd, China. Inhibitors used in this study included the P65 inhibitor Multi-target Pt (#HY-162092),the ERK inhibitor U0126, the W-13 (#HY-13757 A) and dasatinib, all acquired from MCE [54]. The primary antibodies used are listed in Supplemental Table 1.

### Virus packaging and transduction into HCC cells

Lentivirus packaging was performed as previously described [55]. Briefly, plasmids encoding the viral components were co-transfected into HEK-293T cells according to the Effectene Transfection Reagent protocol [55]. Virus-containing supernatants were collected at 48 and 72 h post-transfection. HCC cells were transduced by incubating them overnight with a 1:1 dilution of virus-containing supernatant and complete DMEM, supplemented with 8 µg/mL polybrene. Cells were harvested 72 h later for gene overexpression or knockdown assessment.

### Determination of cell viability, cell proliferation, and cell apoptosis

Following the designated treatments, cell viability, proliferation and apoptosis were measured using MTT, EdU assays, and flow cytometry analysis respectively, following published protocols [56, 57].

### Transwell invasion assay

For the invasion assay, Transwell chambers (BD Biosciences, Franklin Lakes, NJ) with Matrigel-coated membranes were used. A total of 1 × 10^5^ Hep3B or MHCC-97 H cells were seeded in the upper chamber and incubated for 24 h at 37 °C. Cells that had invaded the lower membrane surface were fixed, stained with crystal violet, and counted in five randomly selected fields at 100 × magnification under an optical microscope, following previously established protocols [57].

### Human HCC samples

A total of 18 human HCC tissue samples and matched adjacent non-tumorous specimens utilized to examine WNT7B and SH3GL3 expression were collected from the First Affiliated Hospital of Xi’an Jiaotong University from January 2009 to December 2013. All samples were histopathologically confirmed and their corresponding patients had not received any radiotherapy or chemotherapy before surgery. The collected specimens were stored at − 80 °C.

### Western blot

For protein extraction, 1 × 10^6^ Hep3B and MHCC-97 H cells were lysed using RIPA buffer (Beyotime, Guangzhou, China). Protein concentrations were determined using a BCA protein assay kit (Pierce, Rockford, USA). Western blotting was performed according to previously published methods [55–58].

### In vivo tumorigenesis assays

All animal experiments adhered to protocols approved by the ethical committee of Xi’an Jiao Tong University. In the subcutaneous tumor implantation assay, 1 × 10^6^ Hep3B cells infected with PAQR5 shRNA lentivirus or non-targeting (NT) shRNA were suspended in 100 µL PBS and subcutaneously injected into the left flanks of 4-week-old female BALB/c nude mice (six mice per group). The mice were acquired and housed in the Animal Center at the Medical College of Xi’an Jiao Tong University. Tumor growth was monitored weekly, and tumor volume was calculated using the formula: V (tumor volume: mm3) = 0.5 × [w (width: mm)]^2^ × L (longer diameter: mm). After 21 days, mice were sacrificed, and tumor samples were excised, weighed, fixed, and stained for histological analysis *via* immunohistochemistry, following previously established protocols [56, 57]. The primary antibodies used in immunohistochemistry are listed in Supplemental Table 1 (Additional file 1: Table S1). To evaluate metastatic potential, tail vein injection experiments were performed. Hep3B cells (1 × 10^6^) infected with PAQR5 shRNA lentivirus or NT shRNA, suspended in 100 µL PBS, were injected into the tail veins of nude mice. Six weeks post-implantation, the mice were sacrificed, and their lungs were harvested and paraffin-embedded for hematoxylin and eosin (H&E) staining.

Moreover, an in vivo orthotopic liver tumor model in C57BL/6 mice was established to explore the effect of PAQR5 on the HCC immune microenvironment. Briefly, 1 × 106 Hepa1-6 cells infected with the shVehicle or shPAQR5 lentivirus were suspended 100 µL PBS and subcutaneously injected into the liver of C57BL/6 mice. The mice were sacrificed after four weeks, and the tumors in liver were collected, and then prepared for standard histological detection or western blot analysis.

### T-cell killing assay

After overexpression or depletion of PAQR5 in Hep3B and MHCC-9 H cells, these HCC cells were seeded into a 96-well or 6-well plate and co-cultured for 72 h with human peripheral blood mononuclear cells (PBMCs; #70025, STEMCELL, Vancouver, BC, Canada) that were previously activated with 100 ng/ml anti-CD3 antibody (#317303), 100 ng/ml anti-CD28 antibody (#302913) and 10 ng/ml IL-2 (#589102; BioLegend, San Diego, CA, USA). PBMCs were co-cultured with HCC cells at a ratio of 4:1. Then, HCC cell viability or apoptosis were detected by using MTT assay or flow cytometry analysis.

### Co-culture and expression of IFN-γ and IL-2

HCC cells with PAQR5 knockdown or PAQR5 overexpression were added in 6-well plates (2 × 105cells/well) and cultured overnight. Then, PBMCs were supplemented into 6 well plates respectively at a ratio of 4:1 with respect to attached HCC cells. After co-culture for 72 h, the supernatant from the co-cultures was collected and centrifuged at 14,000 rpm, and then underwent to IFN-γ and IL-2 detection by ELISA assay. The ELISA kits for detection of IFN-γ and IL-2 were purchased from R&D Systems (#: DIF50C for IFN-γ, #: QK202 for IL-2).

### qRT-PCR

After finishing the designed intervention or co-culture, the Trizol Reagent (Thermo Fisher Scientific, California, USA) was used to extract the total RNA from PBMCs, and quantitated the RNA concentration by absorbance at 260 nm. For mRNA detection, the RNA (1 µg) sample was reverse-transcribed using PrimeScript RT Master Mix, and quantitative real-time PCR was performed with SYBR-Green PCR Master Mix (Takara Bio, Dalian, China) using the gene-specific primers. GAPDH was used as loading control, and the results were calculated by the 2^−ΔΔCt^ method.

### m6A RIP-qPCR analysis

1 µg IgG or m6A antibody were incubated with Protein G Magnetic beads in 1x Reaction buffer (150mM NaCl, 10mM Tris-HCl, pH 7.5, 0.1% NP-40 in nuclease free H2O) at 4 °C for 3 h, followed by incubation with 200 µg extracted RNA at 4 °C for 3 h. Incubation of RNA-antibody-conjugated beads with 100 µl Elution Buffer (75 nM NaCl, 50 nM Tris-HCl, pH 7.5, 6.25 nM EDTA, 1% (w/v) SDS, 20 mg/ml Proteinase K) for 30 min at room temperature was utilized to elute the bound RNAs. The eluted RNA was then extracted by phenol: chloroform method followed by ethanol precipitation. Isolated m6A-RIP RNA was reverse transcribed and quantification by qPCR. IP enrichment ratio of a transcript was calculated as the ratio of its amount in IP to that in the input yielded from same amounts of cells. Primer sequences are listed in Supplementary Material 1.

### Statistical analysis

Data were expressed as mean ± S.E.M. Group differences were analyzed using Student’s t-test or one-way ANOVA with post hoc tests, conducted using GraphPad Prism 6 software (GraphPad Software, Inc., La Jolla, CA). Statistical significance was set at *P* < 0.05.

## Results

### Aberrant over-expression and poor prognosis of PAQR5 in HCC

RNA consensus tissue gene data from the Human Protein Atlas (HPA) (https://www.proteinatlas.org/) and Genotype-Tissue Expression (GTEx) project (https://www.gtexportal.org/), using HPA version 23.0 and Ensembl version 109, were analyzed to summarize PAQR5 transcript expression across 50 tissues. The results showed the highest expression levels in liver and bile duct cancers (Fig. S1A).

**Fig. 1:**
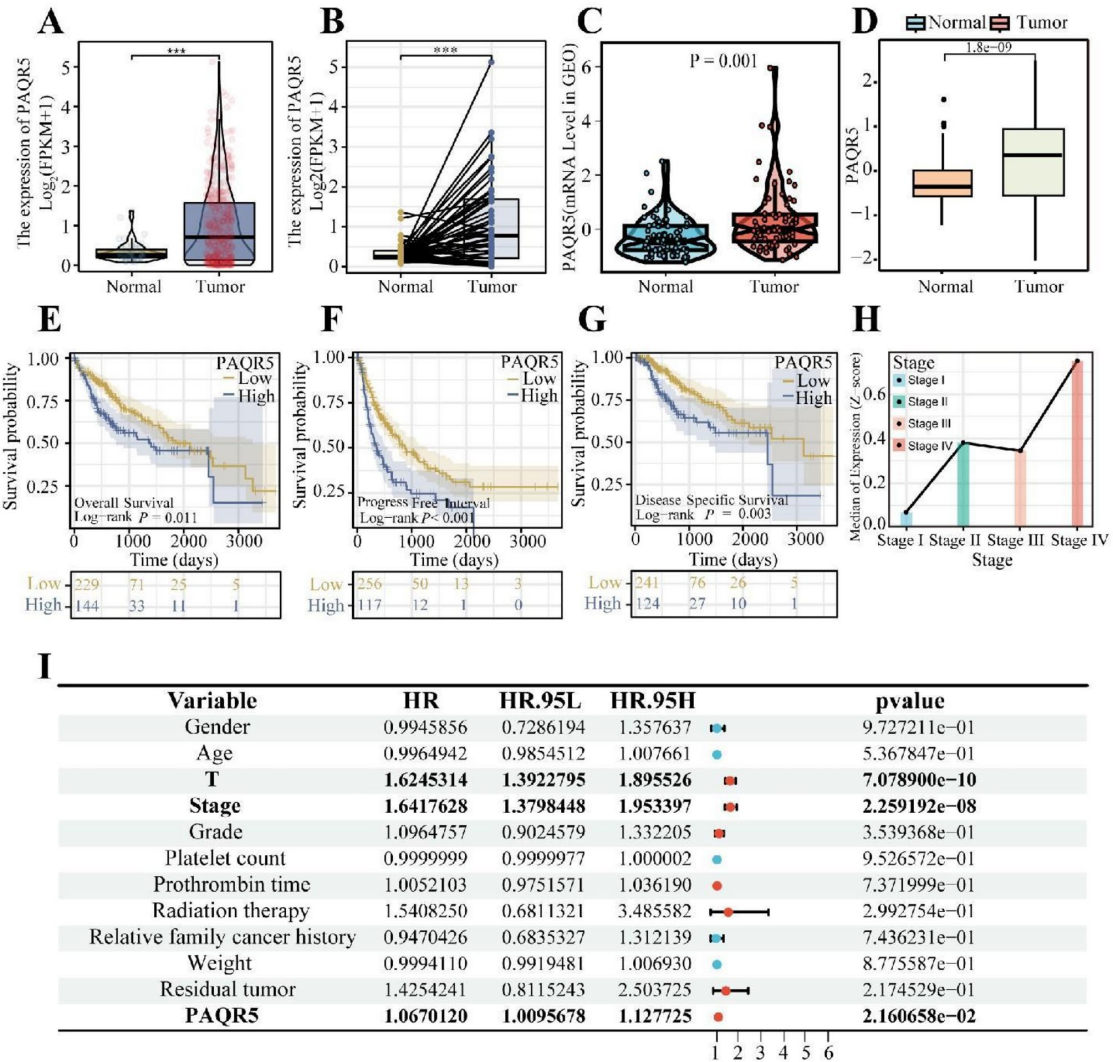
Aberrant over-expression and poor prognosis of PAQR5 in HCC. **A**,** B** The PAQR5 mRNA expression of paired HCC samples and unpaired HCC samples in TCGA-LIHC cohort. **C**,** D** The validation of PAQR5 mRNA expression of HCC samples in GSE39791 cohort and TCGA-GTEx cohort. **E**,** F**,** G** Survival differences between high and low PAQR5 expression groups. Overall survival (OS), progression-free interval (PFI), and disease-specific survival (DSS). **H** Association between HCC Stage and PAQR5 expression. **I** PAQR5 as independent prognostic factors for HCC patients in Cox regression analysis. HR, hazard ratio. *p < 0.05; **p < 0.01; ***p < 0.001

To investigate PAQR5’s role in carcinogenesis, its mRNA expression was examined in HCC datasets from the TCGA and GSE39791 databases. TCGA data analysis revealed significantly higher PAQR5 expression in HCC tissues compared to normal tissues (*P* < 0.001; Fig. 1A). This finding was confirmed through paired analysis, which showed increased PAQR5 expression in HCC tissues relative to adjacent normal tissue (*P* < 0.001; Fig. 1B). Consistent results were observed in independent analyses of the GEO and TCGA-GTEx cohorts, with elevated PAQR5 expression in HCC tissues (*P* = 0.001; Fig. 1C-D). These results suggest that PAQR5 overexpression may be important in HCC development.

### PAQR5 expression serves as an independent prognostic indicator in patients with HCC

Given the elevated expression of PAQR5, this study further explored its clinical significance in HCC. Prognostic and univariate Cox regression analyses were performed in the TCGA cohort. Kaplan-Meier survival curves for overall survival (OS), progression-free interval (PFI), and disease-specific survival (DSS) showed that patients with high PAQR5 expression had significantly poorer survival outcomes compared to those with low expression (OS, *P* = 0.011; PFI, *P* < 0.001; DSS, *P* = 0.003) (Fig. 1E-G). Similar results were found in the GSE54236 cohort (Fig. S1D). Cox regression analysis further identified PAQR5 expression, T stage, and overall staging as independent prognostic factors for HCC (*P* < 0.05, Fig. 1I), indicating that PAQR5 is an independent predictor of prognosis.

Additionally, analysis of clinicopathological characteristics in the TCGA cohort demonstrated that high PAQR5 expression was associated with advanced disease stages (Fig. 1H, Fig. S1B, C, E), reinforcing its potential role in HCC progression and as a prognostic marker.

### PAQR5 is highly enriched in tumor cells to drive tumor progression

**Fig. 2:**
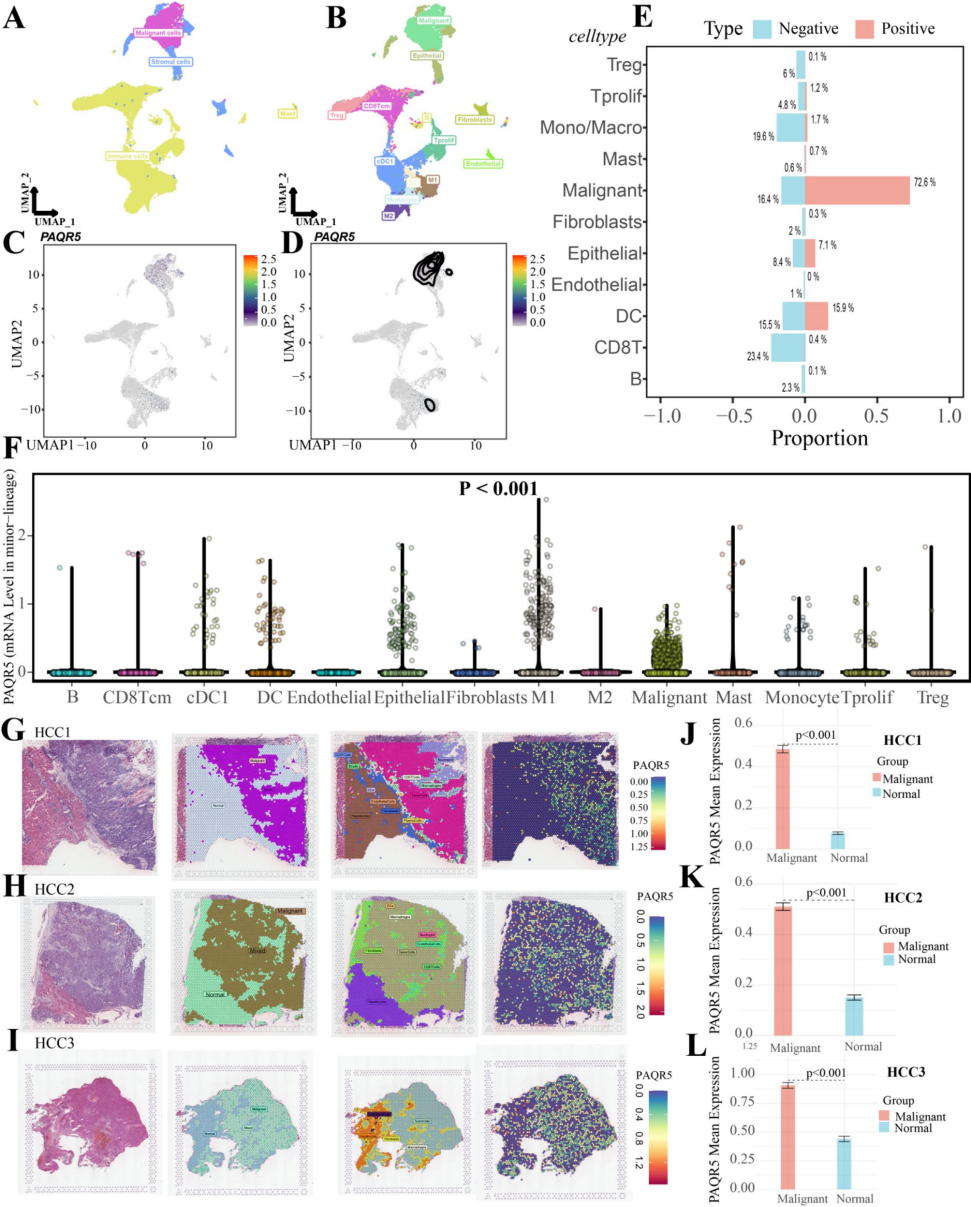
PAQR5 is highly enriched in tumor cells to drive tumor progression. **A**,** B** UMAP plot of broad cell clusters and major cell clusters. **C**,** D** The expression distribution of PAQR5 in HCC cell clusters. **E** The proportion of each cell clusters in the PAQR5 positive/negative group. **F** PAQR5 gene expression across various cell lineages. **G**,** H**,** I** The distribution of spatial tumor micro-environment and PAQR5 expression. **J**,** K**,** L** Differences in PAQR5 expression between malignant and normal regions

In the single-cell transcriptomic analysis, following quality control, batch effect removal, and data standardization, the UMAP plot distinctly visualizes the cell clusters derived from two patients with HCC. The cells were broadly categorized into three main groups: malignant cells, stromal cells, and immune cells (Fig. 2A). Further subdivision revealed major cell clusters, including malignant cells, epithelial cells, fibroblasts, endothelial cells, T regulatory (Treg) cells, CD8 central memory (CD8Tcm) cells, T proliferating (Tprolif) cells, conventional dendritic type 1 (cDC1) cells, dendritic cells, M1 macrophages, M2 macrophages, monocytes, mast cells, and B cells (Fig. 2B).

The UMAP plot highlights significant PAQR5 expression predominantly in malignant tumor cells and M1 macrophages (Fig. 2C and D). This study further calculated the proportions of each cell type within the PAQR5-positive and PAQR5-negative groups, finding a significantly higher proportion of malignant cells in the PAQR5-positive group compared to the negative group (Fig. 2E). Additionally, differential analysis of PAQR5 expression across various cell types confirmed that its expression was primarily confined to malignant cells and M1 macrophages (Fig. 2F). This cell-specific expression pattern suggests that PAQR5 may contribute to HCC progression and play a role in modulating the immune function of macrophages.

In the spatial transcriptomic analysis, the relationship between PAQR5 and the TME was explored. Across all three samples (HCC1, HCC2, HCC3), PAQR5 expression was spatially consistent with the distribution of tumor cells, indicating a predominant expression in the malignant regions (Fig. 2G, H and I). By defining and comparing malignant versus normal regions, significantly higher PAQR5 expression was observed in malignant areas, corroborating previous findings (Fig. 2J, K and L). These results suggest that PAQR5 is closely associated with the tumor cell population within the TME, potentially influencing HCC progression and the local immune landscape.

### PAQR5 shapes the immunosuppressive microenvironment in HCC

**Fig. 3:**
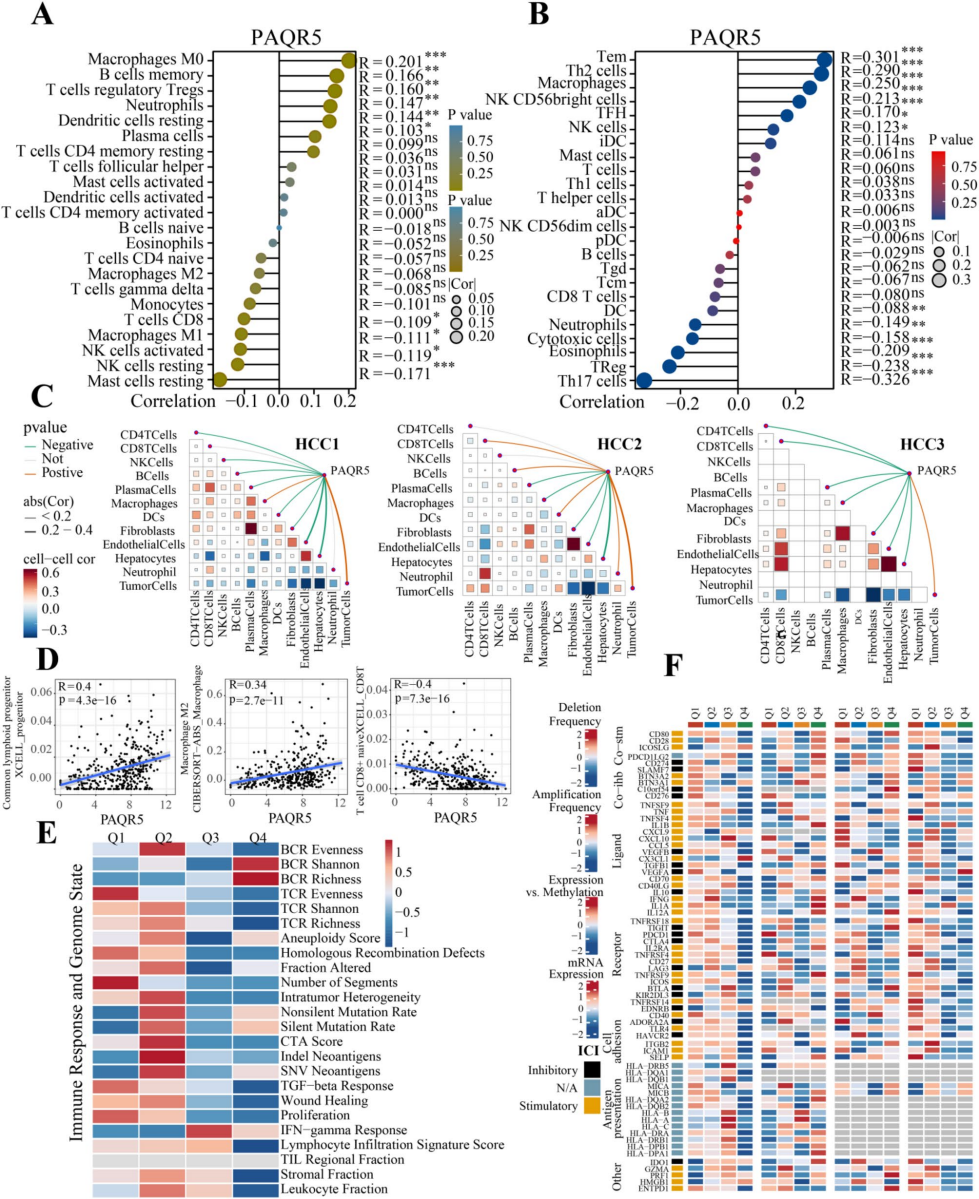
PAQR5 shapes the immunosuppressive microenvironment in HCC. **A**,** B** The correlation between PAQR5 expression and immune infiltration using CIBERSORT and ssGSEA. **C** The spearman correlation of PAQR5 expression with micro-environmental components at spatial resolution. **D** The correlation between PAQR5 and several immune cells using XCELL and CIBERSORT. **E** The relationship between PAQR5 expression and immune response and genome status. **F** The association between immunomodulatory molecules and PAQR5 expression

The correlation between PAQR5 expression and immune infiltration in bulk transcriptomic data was assessed using CIBERSORT (Fig. 3A) and ssGSEA (Fig. 3B). Immune cell types positively correlated with PAQR5 expression included M0 macrophages, memory B cells, regulatory T cells (Tregs), neutrophils, resting dendritic cells, plasma cells, effector memory T cells (Tem), Th2 cells, macrophages, NK CD56bright cells, T follicular helper (TFH) cells, and NK cells. In contrast, negative correlations were observed with resting mast cells, resting NK cells, activated NK cells, M1 macrophages, CD8 T cells, Th17 cells, Tregs, eosinophils, cytotoxic cells, neutrophils, and dendritic cells. These results were validated across additional cohorts (Fig. S2B).

In the spatial transcriptomic analysis, the Spearman correlation between PAQR5 expression and microenvironmental components at spatial resolution was visualized (Fig. 3C). Consistent with previous localization results, PAQR5 expression showed a significant positive correlation with the abundance of malignant cells, while displaying a significant negative correlation with several anti-tumor immune cells, including CD4 T cells, NK cells, and B cells in HCC1, as well as macrophages in HCC2 and HCC3.

Further investigation of PAQR5 correlations with specific immune cells using XCELL and CIBERSORT (Fig. 3D) revealed a positive correlation with common lymphoid progenitor cells (*R* = 0.4, *P* = 4.3e-16) and M2 macrophages (*R* = 0.34, *P* = 2.7e-11), while a negative correlation was identified with naïve CD8 T cells (*R* = -0.4, *P* = 7.3e-16), indicating an association between PAQR5 expression and an immunosuppressive microenvironment.

The relationship between PAQR5 expression, immunogenicity scores, and DNA damage was also explored to elucidate its impact on immune response and genomic integrity (Fig. 3E). Given the critical role of immunomodulatory molecules in cancer immunotherapy, the association between these molecules and PAQR5 expression was examined to construct a comprehensive immune landscape related to PAQR5 (Fig. 3F, S2A).

### Enrichment analysis and hallmark functions of PAQR5 in HCC

**Fig. 4:**
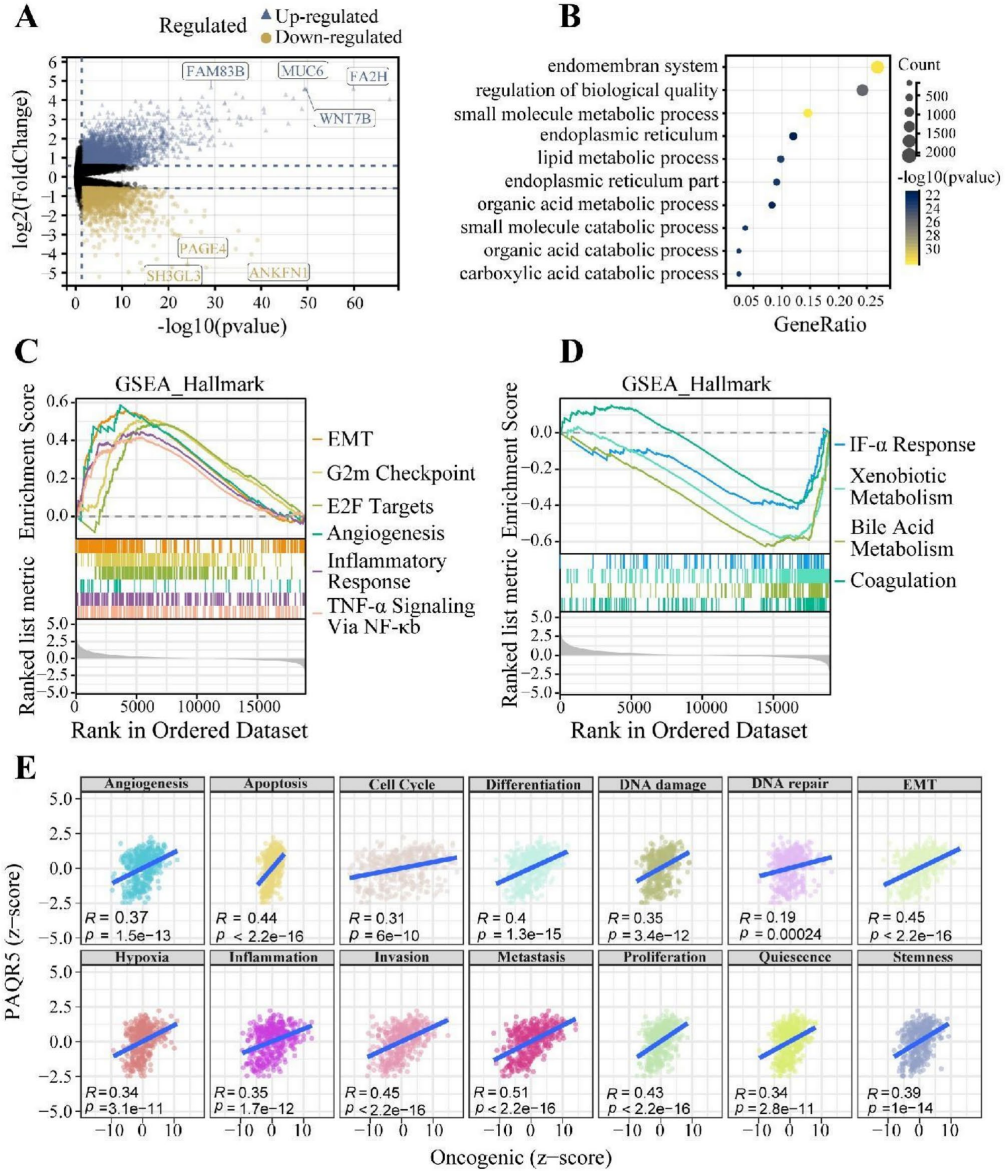
Enrichment analysis and function hallmark of PAQR5 in HCC. **A** Differentially expressed genes. **B** The results of KEGG enrichment analysis. **C**,** D** GSEA based on the Hallmark gene sets. **E** The correlation between cancer-related phenotypes and PAQR5 expression by GSVA. EMT, Epithelial-mesenchymal transition. *p < 0.05; **p < 0.01; ***p < 0.001

To elucidate the biological function of PAQR5 in HCC, samples were stratified into two groups based on the median PAQR5 expression level for differential gene expression analysis. This analysis identified significantly upregulated and downregulated genes, using thresholds of|log2 Fold change|> 1 and *P* < 0.05 (Fig. 4A). Consistently, western blot analysis also confirmed that WNT7B were notably upregulated in human HCC tissues, while SH3GL3 was greatly decreased in HCC specimens compared with matched adjacent non-malignant tissues (Fig S3).

Subsequent KEGG enrichment analysis revealed that these genes were mainly involved in processes such as the endomembrane system, regulation of biological quality, small molecule metabolism, endoplasmic reticulum functions, lipid metabolism, organic acid metabolism, and carboxylic acid catabolism (Fig. 4B). Additionally, GSEA using Hallmark gene sets indicated that pathways related to epithelial-mesenchymal transition (EMT), G2/M checkpoint, E2F targets, angiogenesis, inflammatory response, and TNF-α signaling *via* the NF-κB pathway were upregulated in the PAQR5 high-expression group (Fig. 4C). In contrast, pathways associated with interferon-α response, xenobiotic metabolism, bile acid metabolism, and coagulation were downregulated in this group (Fig. 4D). Further GSEA validation across multiple HCC cohorts consistently demonstrated significant activation of the NF-κB pathway in those with high PAQR5 expression (Fig. S4A).

Gene Set Variation Analysis (GSVA) was performed to assess the relationship between PAQR5 expression and cancer-related phenotypes. Results showed that PAQR5 expression significantly influenced various tumor-related phenotypes, including angiogenesis (*R* = 0.37, *P* = 1.5e-13), apoptosis (*R* = 0.44, *P* < 2.2e-16), cell cycle regulation (*R* = 0.31, *P* = 6e-10), cellular differentiation (*R* = 0.4, *P* = 1.3e-15), DNA damage response (*R* = 0.35, *P* = 3.4e-12), DNA repair (*R* = 0.19, *P* = 0.00024), EMT (*R* = 0.45, *P* = 2.2e-16), hypoxia response (*R* = 0.34, *P* = 3.1e-11), inflammation (*R* = 0.35, *P* = 1.7e-12), invasion (*R* = 0.45, *P* < 2.2e-16), metastasis (*R* = 0.51, *P* < 2.2e-16), proliferation (*R* = 0.43, *P* < 2.2e-16), quiescence (*R* = 0.34, *P* = 2.8e-11), and stemness (*R* = 0.39, *P* = 1e-14) (Fig. 4E).

### PAQR5 drives heterogeneity in single-cell cell communication landscapes

**Fig. 5:**
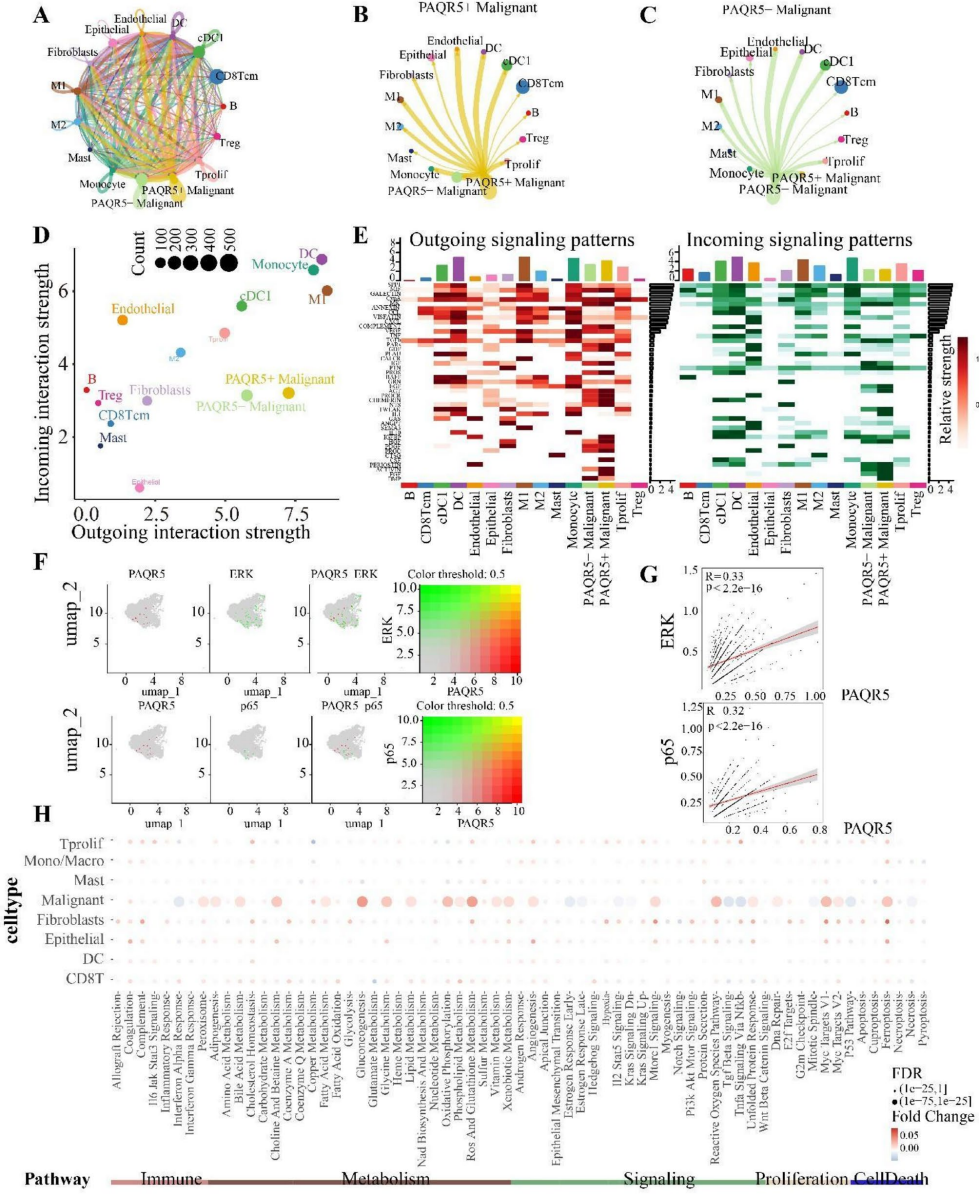
PAQR5 drives heterogeneity in single-cell cell communication landscapes. **A** Cellular communication between different cell lineages. **B**,** C** the cellular communication landscapes of PAQR5 + Malignant and PAQR5-Malignant with other cell lineages. **D** The similarities of the communication landscapes of different cell clusters. **E** Those communication signaling molecules with the highest weights in each cluster. **F**,** G** The activation of the NF-κB pathway in malignant cell cluster. **H** The activation of some biological pathways among different cell clusters. *p < 0.05; **p < 0.01; ***p < 0.001

Using the CellChat method, the cellular communication networks among different cell lineages in the GSE166635 dataset were visualized (Fig. 5A). The analysis specifically highlighted the communication landscapes between PAQR5 + malignant cells and PAQR5- malignant cells in relation to other cell clusters (Fig. 5B and C). Comparative analysis of these communication networks revealed that while PAQR5 + malignant cells and PAQR5- malignant cells exhibited similar levels of received signaling, PAQR5 + malignant cells demonstrated significantly higher levels of outgoing (sent) signals, indicating a potential role of PAQR5 in enhancing tumor cell-secreted signaling (Fig. 5D). The communication signaling molecules with the highest interaction weights were identified and visualized for each cluster, highlighting key differences in the communication landscapes (Fig. 5E).

In single-cell transcriptomics, subsequent to the previously described enrichment analyses, the activation of the NF-κB pathway was further examined within the malignant cell cluster. It was found that critical molecules involved in the NF-κB pathway, including ERK and p65, were significantly co-activated with PAQR5 in malignant tumor cells (Fig. 5F and G). The AUCell package was employed to evaluate the activation of various biological pathways across different cell clusters, revealing significant alterations in NF-κB pathway activation within the malignant cell clusters, consistent with prior analyses (Fig. 5H).

### Epigenetic and genomic mechanisms of dysregulated PAQR5 expression

The dysregulation of PAQR5 expression appears to be driven by epigenetic mechanisms. Analysis of methylation levels in the 5’ untranslated region (5’ UTR) of PAQR5 revealed significantly higher methylation in normal tissues compared to tumor tissues, suggesting that hypermethylation may contribute to PAQR5 silencing and reduced expression in normal cells (Fig. S5A). We also performed m6A-RIP-qPCR assay to further detect the m6A level of 5’ UTR-PAQR5 mRNA in HCC. The m6A-RIP-qPCR verified that the high m6A antibody enriched in 5’ UTR-PAQR5 mRNA of matched adjacent non-malignant tissues and normal human liver cell line: MIHA cells, while this enrichment significantly reduced in HCC specimens, Hep3B and MHCC-97 H cells (Fig S6A, S6B). RNA editing data obtained from the SYNAPSE platform (https://www.synapse.org/) indicated that the RNA editing level at the chr15|6,969,965|UTR3|PAQR5|+|Alu|chimp locus was higher in tumor tissues, potentially influencing PAQR5 regulation in cancer (Fig. S5B). Additionally, genetic causal variation between PAQR5 and HCC was identified, further implicating PAQR5 in tumor development (Fig. S5C).

ATAC-seq analysis identified five peaks associated with PAQR5 transcription, spanning from 3000 base pairs upstream to 3000 base pairs downstream of the transcription start site, which may indicate regions involved in the regulation of PAQR5 expression (Fig. S5G). Spearman correlation analysis revealed significant associations between multiple transcription factors and ATAC-seq peaks, with different patterns observed across various chromosomal regions. In certain regions, transcription factor expression levels were positively correlated with ATAC peak intensity, suggesting a role in maintaining open chromatin at these location (Fig. S5D). The types of peak locations were classified, encompassing Promoter, 5’ UTR, 3’ UTR, Exon, Intron, Downstream, and Intergenic regions, providing insight into the potential regulatory elements affecting PAQR5 (Fig. S5E, S5F). Genome copy number variation (CNV) analysis, based on GISTIC scores, was conducted using data from 370 samples, revealing multiple chromosomal CNVs in the TCGA-LIHC cohort, which may contribute to altered PAQR5 expression in HCC (Fig. S5H). Specifically, we evaluated the correlation coefficients between PAQR5 copy number scores calculated by Gistic2 and PAQR5 mRNA expression in TCGA-LIHC cohort. The results showed a non-significant correlation, indicating that the aberrant upregulation of PAQR5 expression cannot be attributed to copy number variation (Fig. S5I).

W-13 was identified as a potential agent capable of reversing the molecular alterations associated with dysregulated PAQR5 expression, effectively counteracting its pro-oncogenic effects (Fig. S4B). A negative correlation was observed between PAQR5 expression and the AUC value of the tyrosine kinase inhibitor dasatinib, indicating that higher levels of PAQR5 corresponded to increased sensitivity to chemotherapeutic agents (Fig. S4C). To further validate the effects of W-13 and dasatinib on the PAQR5 expression in HCC, we used the W-13 and dasatinib to intervene HCC cells, and the western blot analysis substantiated that both W-13 and dasatinib effectively downregulated the PAQR5 expression in HCC cells (Fig S6C, S6D).

### PAQR5 is significantly elevated in HCC cell lines

Western blot analysis revealed significantly elevated PAQR5 expression in HCC cell lines compared to the immortalized normal hepatic cell line MIHA (*P* < 0.05, Fig. S8). Based on these findings, Hep3B and MHCC-97 H cell lines were selected for subsequent functional assays.

**Fig. 6:**
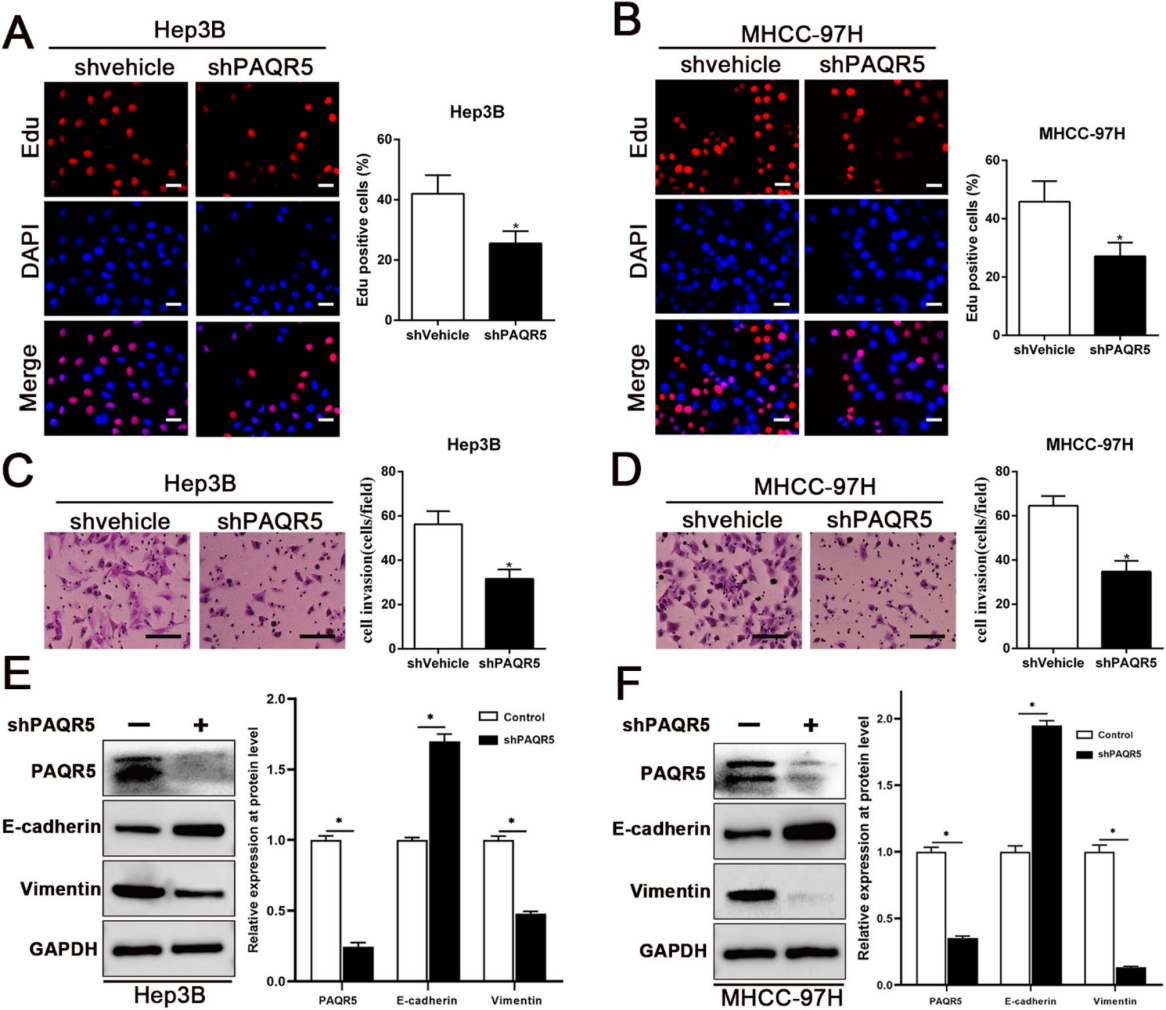
Targeting PAQR5 repressed the proliferation, invasion and EMT of HCC cells. **A**,** B**,** C**,** D**,** E**,** F** MTT, Edu, transwell and WB analysis were conducted to assess the efficacy of PAQR5 on cell viability, proliferation, invasion and EMT in HCC. Magnification of Edu is ×200, and scale bars = 50 μm; and Magnification of Transwell is ×400, and scale bars = 20 μm. *p < 0.05

### Targeting PAQR5 repressed the proliferation, invasion, and EMT of HCC cells

To investigate the effects of PAQR5 on HCC cells, PAQR5 shRNA lentivirus was used to knock down PAQR5 expression in Hep3B and MHCC-97 H cells. As shown in Fig. 6A-F and Fig. S7, PAQR5 levels were significantly reduced following shRNA transfection (*P* < 0.05). Functional assessments, including MTT, EdU, transwell, and Western blot analyses, demonstrated that PAQR5 depletion led to marked reductions in cell viability, proliferation, invasion, and EMT (*P* < 0.05), thereby supporting PAQR5’s role in promoting oncogenic processes in HCC.

### Targeting PAQR5 inhibited tumorigenicity and invasion-metastasis cascades in vivo

**Fig. 7:**
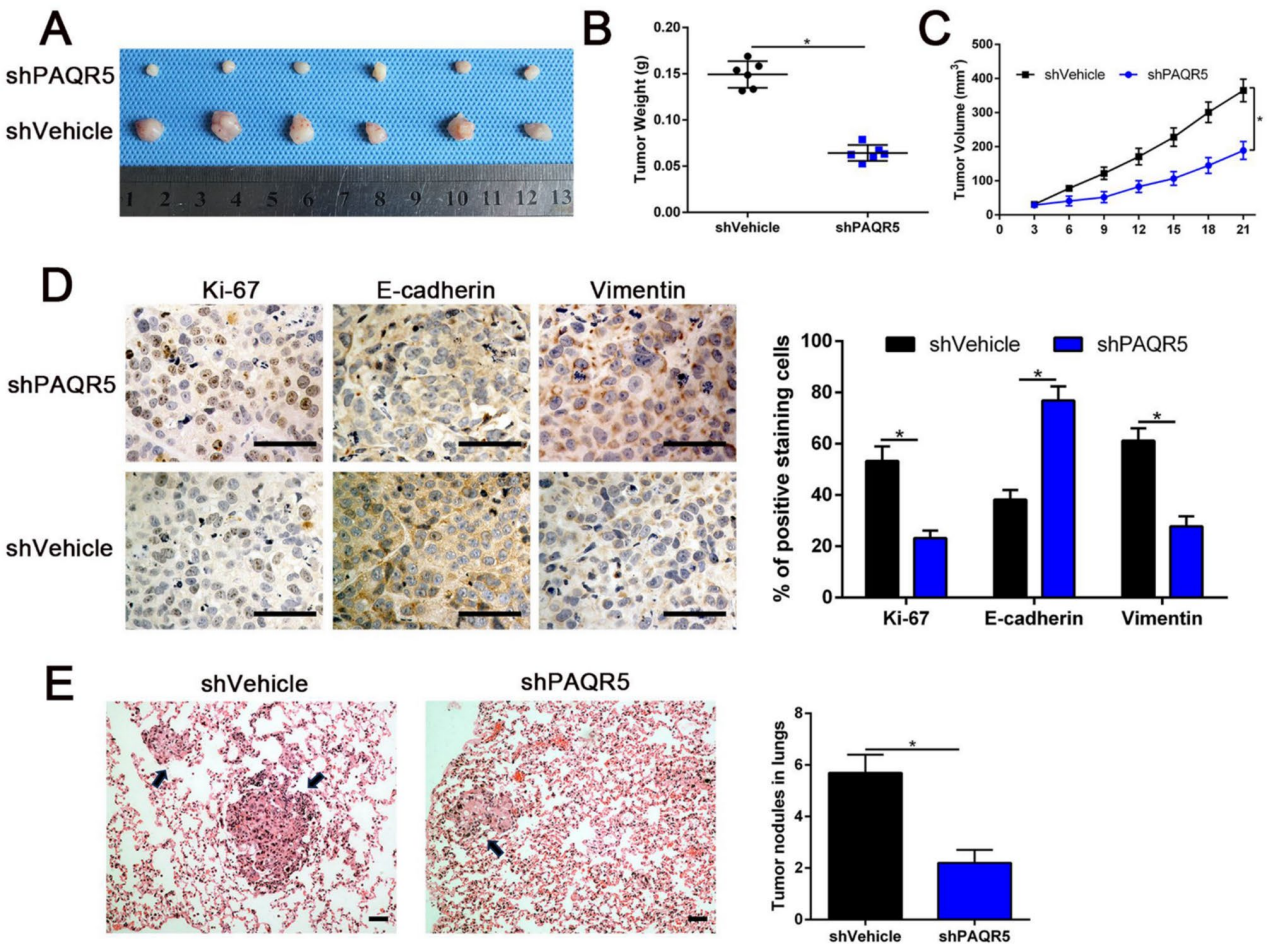
Targeting PAQR5 inhibited tumorigenicity and invasion-metastasis cascades in vivo. **A**,** B**,** C** PAQR5 knockdown effectively inhibited HCC tumor growth compared with vehicle control group. **D** Immunohistochemical analysis of subcutaneous tumor tissues from PAQR5 knockdown group compared to samples from control group. Magnification is × 400, the scale bar represents 20 μm, *n* = 6. **E** HE staining of dissected lungs from sacrificed mice verified less and smaller metastatic nodules caused by PAQR5 knockdown. Magnification is × 100, the scale bar represents 50 μm. *n* = 6, *p < 0.05

To evaluate the impact of PAQR5 on the tumorigenic potential of HCC cells in vivo, Hep3B cells with PAQR5 knockdown were subcutaneously injected into the flanks of nude mice to establish a xenograft tumor model. In vivo analysis of these subcutaneous xenografts confirmed the in vitro findings, showing that PAQR5 knockdown significantly inhibited tumor growth compared to the vehicle control group (*P* < 0.05, Fig. 7A–C). Immunohistochemical staining demonstrated that tumor tissues from the PAQR5 knockdown group exhibited reduced Ki-67 and vimentin expression, alongside increased E-cadherin staining, compared to controls (*P* < 0.05, Fig. 7D).

Additionally, a lung metastasis model was established in nude mice to investigate the role of PAQR5 in HCC pulmonary colonization. Six weeks following the tail vein injection of PAQR5-depleted Hep3B cells, mice were sacrificed, and lung tumor nodules were examined. PAQR5 knockdown markedly reduced the metastatic capacity of Hep3B cells in vivo. HE staining of lung tissues confirmed fewer and smaller metastatic nodules in the PAQR5 knockdown group compared to controls (*P* < 0.05, Fig. 7E). Overall, these in vivo findings support the in vitro data, indicating that targeting PAQR5 effectively suppresses the proliferation, EMT, and metastasis of HCC cells.

### PAQR5 activated the NF-κB signaling by inducing the phosphorylation of ERK

**Fig. 8:**
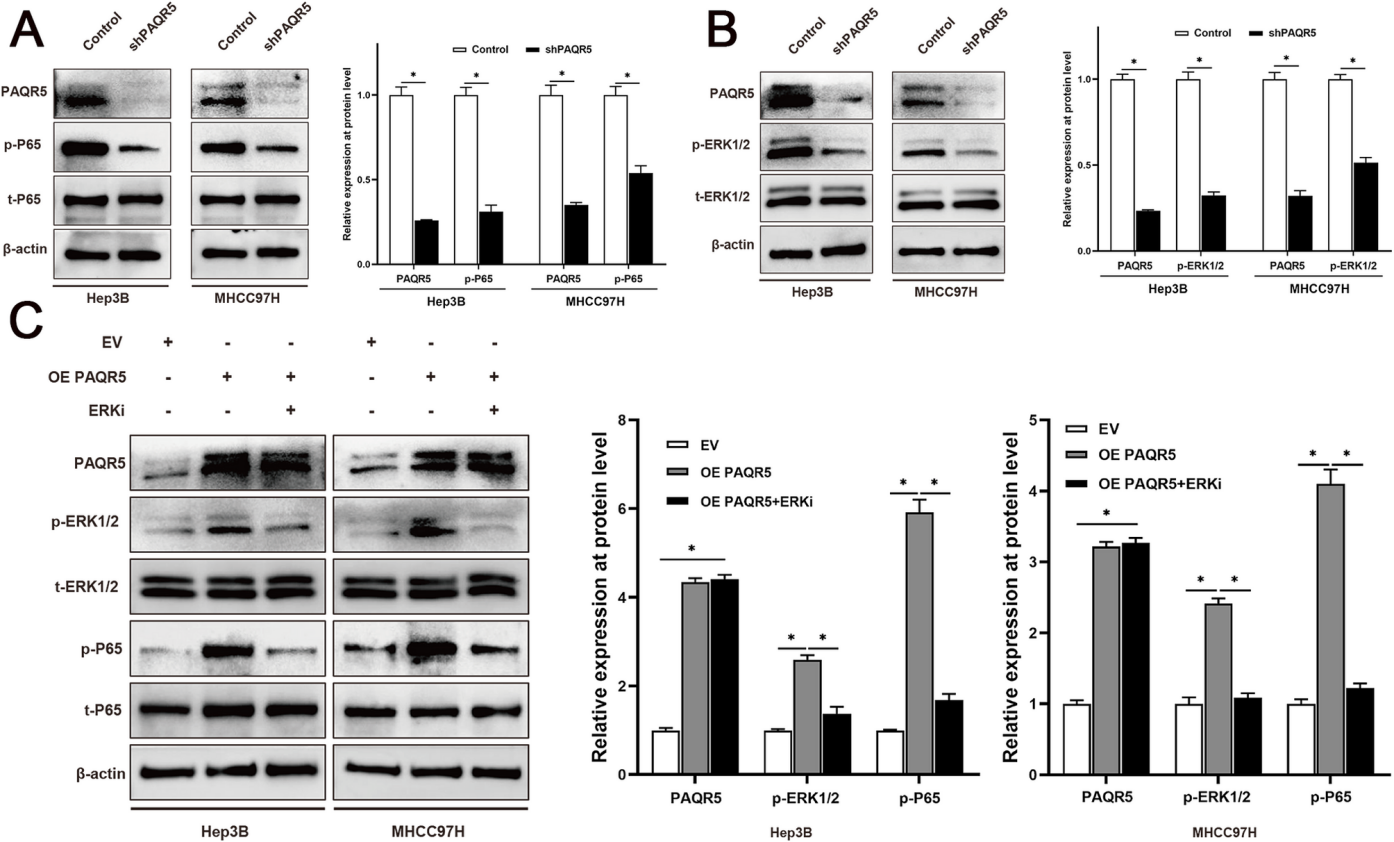
PAQR5 activated the NF-κB signaling through inducing the phosphorylation of ERK. **A** Depletion of PAQR5 in HCC cells significantly decreased the phosphorylation of p65. **B** Knockdown of PAQR5 prominently decreased the p-ERK and over-expression of PAQR5 greatly increased the p-ERK level. **C** Over-expression of PAQR5 in HCC cells significantly elevated the p-p65 level as well, whereas targeting ERK signaling by ERK inhibitor U0126 notably abrogated the over-expression of PAQR5 induced the increase the phosphorylation of p65. *p < 0.05

To elucidate the molecular mechanisms by which PAQR5 promotes proliferation, EMT, and metastasis in HCC, bioinformatics analysis was performed, indicating that PAQR5 modulates the NF-κB signaling pathway. Accordingly, PAQR5 was knocked down in HCC cells, and Western blot analysis was used to measure the expression of phosphorylated p65 (p-p65, Ser-536). Results showed that PAQR5 depletion significantly reduced p65 phosphorylation (*P* < 0.05, Fig. 8A). Previous studies have established that activation of enteroendocrine membrane progesterone receptors markedly increases p-ERK levels [59], with p-ERK serving as a key upstream regulator of p-p65 [60, 61]. Therefore, the role of ERK signaling in PAQR5-mediated NF-κB activation was further investigated. Knockdown of PAQR5 substantially decreased p-ERK levels, while PAQR5 overexpression significantly elevated p-ERK (*P* < 0.05, Fig. 8B and C). Additionally, PAQR5 overexpression increased p-p65 levels, but this effect was notably diminished by the ERK inhibitor U0126, which blocked the PAQR5-induced phosphorylation of p65 (*P* < 0.05, Fig. 8C). These results suggest that PAQR5 activates NF-κB signaling through ERK phosphorylation.

### PAQR5 promoted the proliferation, invasion, and EMT of HCC cells through the activation of NF-κB signaling

**Fig. 9:**
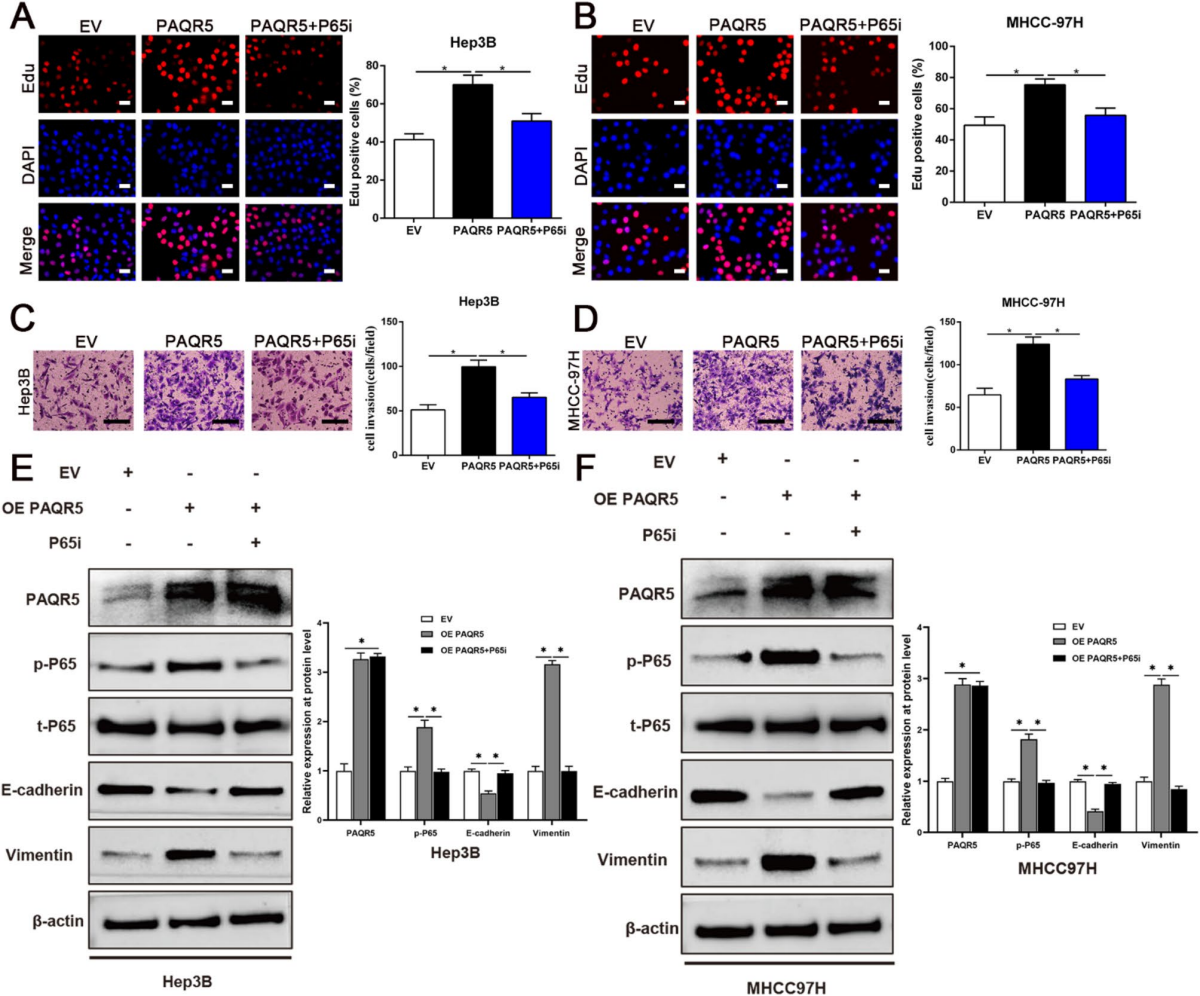
PAQR5 promoted the proliferation, invasion and EMT of HCC cells through activation of NF-κB signaling. **A**,** B**,** C**,** D**,** E**,** F** Over-expression of PAQR5 greatly facilitated the viability, proliferation, invasion and EMT of HCC cells, while inhibiting the phosphorylation of p65 could notably mitigate the overexpression of PAQR5 induced the promotion of cell viability, proliferation, invasion and EMT in HCC. Magnification of Edu is ×200, and scale bars = 50 μm; and Magnification of Transwell is ×400, and scale bars = 20 μm. *p < 0.05

To further examine the role of the PAQR5/p65 axis in HCC cell proliferation, invasion, and EMT, rescue assays were conducted. As shown in Fig. 9A–F, PAQR5 overexpression significantly enhanced cell viability, proliferation, invasion, and EMT, whereas inhibition of p65 phosphorylation effectively attenuated these PAQR5-induced effects (*P* < 0.05). These results demonstrate that PAQR5 facilitates HCC progression by activating the NF-κB signaling pathway, driving oncogenic processes such as proliferation, invasion, and EMT.

### PAQR5 induces tumor immune escape in HCC by promoting PD-L1 expression through activating ERK/NF-κB signaling

The expression level of PD-L1 is a determining factor affecting the formation of immunosuppressive microenvironment in HCC. To explore whether PAQR5 mediates the immune escape of HCC through inducing PD-L1, we conducted western blot analysis to examine the PD-L1 expression level after knockdown PD-L1 in HCC cells. As displayed in Fig S9, depletion of PAQR5 in Hep3B, MHCC-97 and Hepa 1–6 significantly reduced the expression of PD-L1 compared with control group. Moreover, PAQR5 overexpression induced the upregulation of PD-L1, but this effect was obviously mitigated by the ERK or p65 inhibitor (*P* < 0.05, Fig. S9). These results suggest that PAQR5 induces PD-L1 expression through activating ERK/NF-κB signaling.

### PAQR5 suppresses anticancer immunity in HCC

**Fig. 10:**
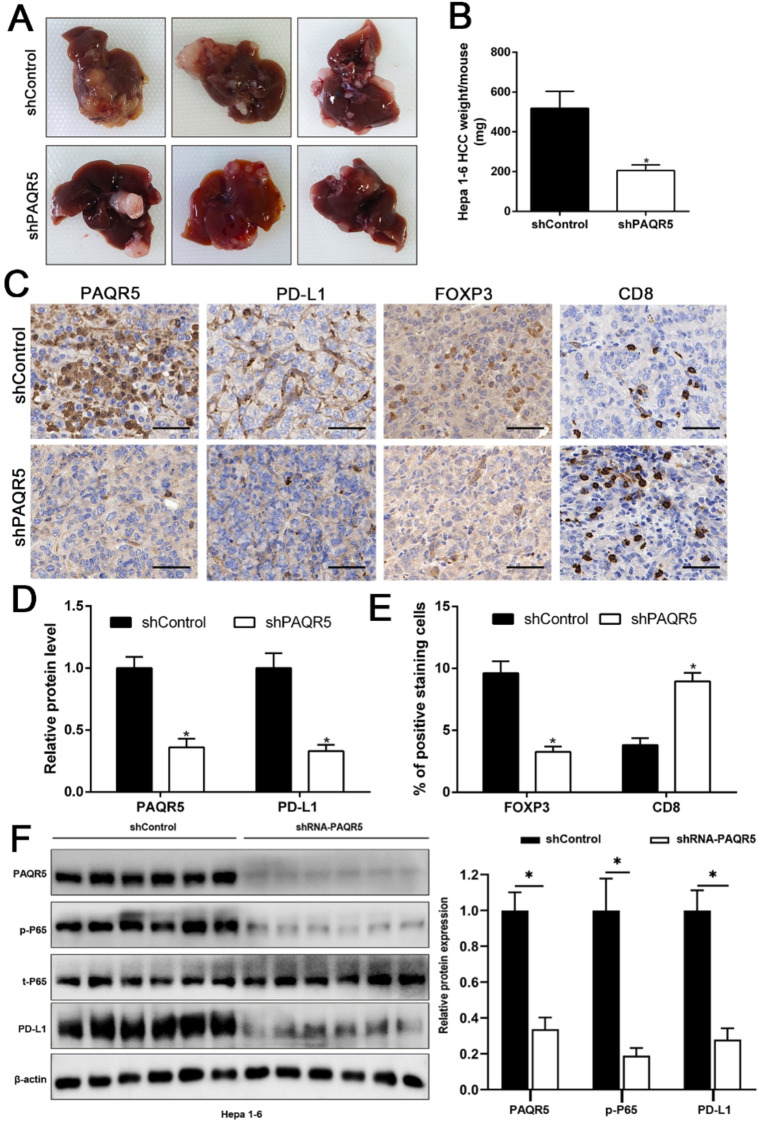
Targeting PAQR5 in HCC cells impeded the formation of the immunosuppressive microenvironment in vivo. **A-B.** the orthotopic liver tumor model in immune-competent C57BL/6 mice validated that knockdown of PAQR5 in mouse cell line: Hepa 1–6 notably inhibited the tumorigenesis and growth. **C-E.** IHC staining revealed that depletion of PAQR5 in Hepa 1–6 prominently repressed the expression of PD-L1, inhibited infiltration of FOXP3 + Treg cells, while facilitated the CD8 + T cell infiltration. **F.** WB performed with tumor lysates revealed that the protein level of p-P65 and PD-L1 was significantly reduced in liver tumors with Hepa 1–6 knockdown than in those tumors in shControl group. Magnification 20x, scale equal to 50 μm. *p < 0.05

To investigate the effect of PAQR5 on the tumor immune microenvironment, the T-cell-mediated killing assay was performed to assess whether the upregulation of PAQR5 in HCC affects T-cell function. In the HCC cells-PBMC co-culture system, PBMCs repressed the HCC cell viability and promoted apoptosis in control group (Fig S10A-B, Fig S11A-B); overexpression of PAQR5 in HCC cells reversed the repressive effects of PBMCs on cell viability, whereas weakened the facilitation effect on apoptosis (Fig S10A-B). However, knockdown PAQR5 in Hep3B and MHCC-97 H cells had the contrary effect. Depletion of PAQR5 in HCC cells increased the repressive effects of PBMCs on cell viability, and enhanced the facilitation effect on apoptosis (Fig S11A-B). The cytotoxic T-cell activity of PBMCs was decreased in co-cultures of PAQR5-overexpressed HCC cells compared with co-cultures with HCC EV cells (Fig S10C-E). On the contrary, the cytotoxic T-cell activity of PBMCs was prominently elevated after co-cultures with PAQR5-depleted HCC cells (Fig S11C-E). Additionally, the orthotopic liver tumor model in immune-competent C57BL/6 mice was established to explore the effect of PAQR5 on the HCC immunosuppressive microenvironment in vivo. After the transplanted Hepa 1–6 cells grew in situ for four weeks, the C57BL/6 mice were sacrificed and the tumor nodules were detected. Depletion of PAQR5 notably impeded the growth of HCC cells in vivo (Fig. 10). Furthermore, IHC staining and western blot analysis verified less PD-L1 expression and FOXP3 + Treg cells but more CD8 + cells in mice tumor tissue caused by PAQR5 knockdown (*P* < 0.01, Fig. 10). Thus, these results offer in vivo support to our in vitro results that PAQR5 can effectively induce the formation of immunosuppressive microenvironment in HCC.

## Discussion

HCC is the most common form of primary liver cancer, characterized by high mortality and recurrence rates [62]. Elucidating the molecular mechanisms underlying hepatocarcinogenesis is crucial for advancing therapeutic strategies and identifying reliable prognostic biomarkers. PAQR5, a member of the progesterone and adiponectin Q receptor family and a subtype of mPR, has been primarily studied for its role in reproductive endocrine signaling and neurodevelopment [63, 64]. Recent evidence suggests that PAQR5 functions as a prognostic marker and protective factor in esophageal adenocarcinoma [65], and it plays a significant role in the antitumor activity of progesterone in ovarian and endometrial cancers, where elevated PAQR5 expression correlates with a lower FIGO stage [15, 19]. However, a systematic investigation of PAQR5 expression in HCC and its clinical prognostic implications is lacking. This study addresses this gap by employing a comprehensive approach that integrates bioinformatics, functional assays in vitro and in vivo, and analysis of clinical samples to delineate the role of PAQR5 in HCC. The aim is to clarify PAQR5’s prognostic significance, explore its multi-level molecular mechanisms in HCC, and establish a foundation for novel therapeutic strategies targeting this receptor. This investigation represents the first extensive examination of PAQR5 in HCC, potentially paving the way for innovative clinical treatment approaches.

**Fig. 11:**
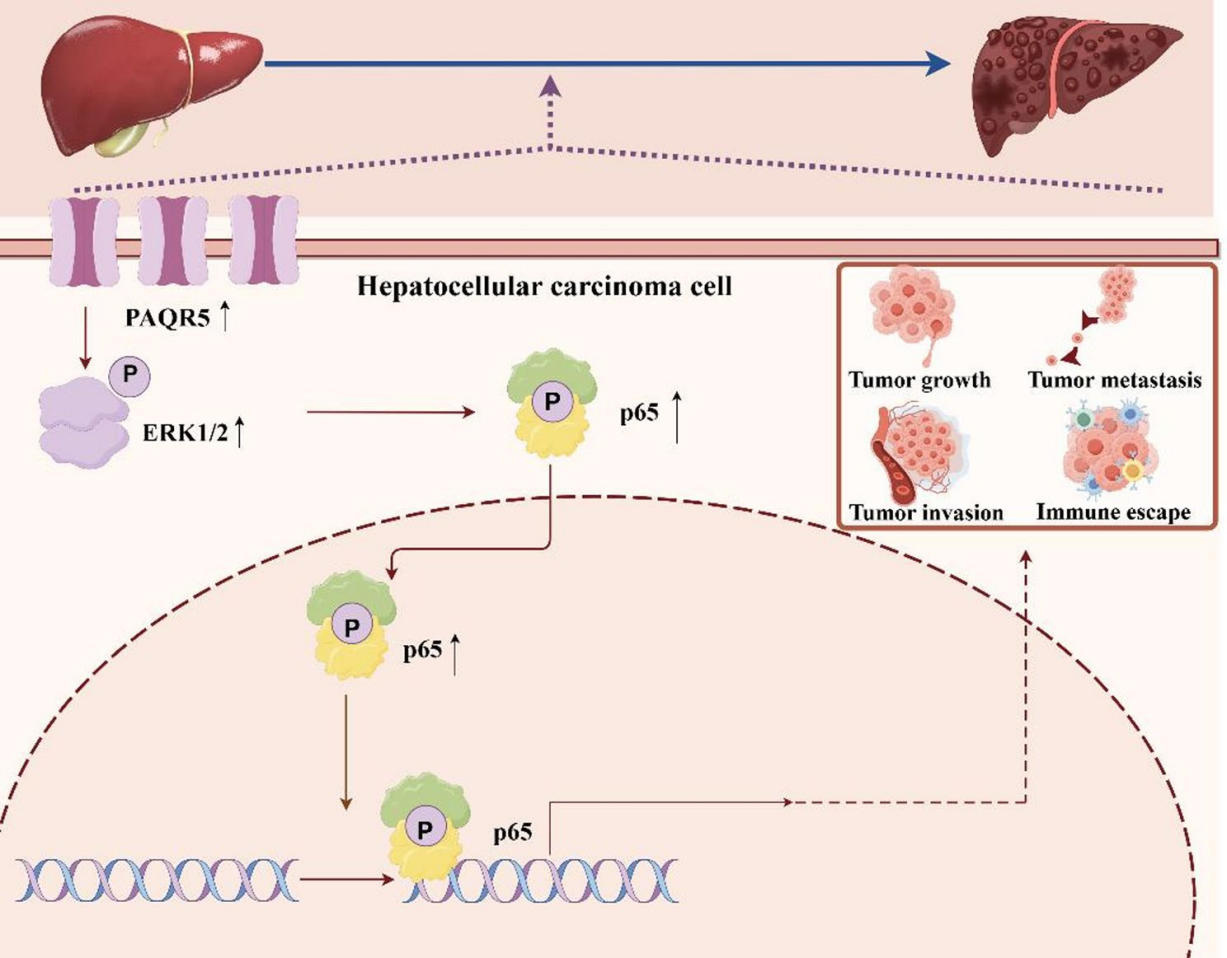
Schematic findings of the study

This study investigated the role of PAQR5 in HCC, utilizing clinical data and RNA-seq analysis to demonstrate that PAQR5 expression is significantly elevated in HCC tissues compared to normal counterparts. Prognostic evaluations revealed that increased PAQR5 expression correlates with poorer patient outcomes, establishing its independent prognostic significance in HCC. Analysis of immune infiltration, spatial expression patterns, functional pathways, and associated phenotypes indicated that PAQR5 could serve as a valuable diagnostic and prognostic biomarker, with its expression levels showing a positive correlation with tumor-infiltrating immune cells and related marker genes. Additionally, several molecular pathways potentially mediated by PAQR5 were identified. Subsequent in vitro and in vivo experiments validated the bioinformatics findings, confirming PAQR5 as an independent prognostic factor with high diagnostic accuracy in HCC. The study’s schematic findings are illustrated in Fig. 11. Nevertheless, further research is required to clarify the mechanisms driving PAQR5 expression and its functional impact on cancer progression.

The immune microenvironment encompasses the interactions between immune cells, inflammatory mediators, cytokines, and other immune-related elements within the tumor microenvironment [66]. In HCC, this environment typically alternates between immunosuppressive and immune-activated states. The immunosuppressive state is characterized by the presence of regulatory T cells (Tregs), tumor-associated macrophages (TAMs), and immunosuppressive cytokines such as TGF-β and IL-10 [67, 68]. In contrast, immune activation is marked by active immune cells, including cytotoxic T lymphocytes (CTLs) and natural killer (NK) cells [69, 70]. This study explored the association between PAQR5 expression and immune cell infiltration in HCC using CIBERSORT and ssGSEA analyses. The results revealed a significant correlation between PAQR5 expression and various immune cell types, suggesting that PAQR5 may play a role in modulating the immune landscape of HCC. PAQR5 expression was positively correlated with M0 macrophages, M2 macrophages, Tregs, and resting dendritic cells. M0 macrophages, representing an undifferentiated state, can differentiate into either tumor-promoting M2 macrophages or anti-tumor M1 macrophages. Elevated M0 macrophage levels may indicate a pro-inflammatory tumor microenvironment linked to increased tumor progression risk [71, 72]. Additionally, Wnt ligands derived from cells have been shown to facilitate M2 macrophage polarization into TAMs *via* the Wnt/β-catenin pathway, while inhibiting Wnt signaling or activating the pathway in TAMs can help control HCC progression [73]. Tregs, known for their role in immune escape, are associated with poor clinical outcomes, suggesting that PAQR5 may contribute to an immunosuppressive microenvironment that fosters tumor growth [74]. Conversely, PAQR5 expression was negatively correlated with resting mast cells, activated NK cells, and CD8 + T cells. As key mediators of anti-tumor immunity, CD8 + T cells, or CTLs, are linked to improved survival in HCC [75–78]. However, elevated PAQR5 expression may suppress this cytotoxic immune response, as HCC-specific cell cycle-associated kinases can enhance IL-6 production and activate nuclear factor-κB (NF-κB) through zeste homolog 2 (EZH2), leading to an accumulation of polymorphonuclear myeloid-derived suppressor cells (MDSCs) and inhibition of T-cell activity [79]. The inverse relationship between PAQR5 and CTLs suggests a diminished immune response associated with poor prognosis. These results align with previous studies, indicating that PAQR5 may be a promising therapeutic target. Further research is warranted to clarify its role in immunomodulation and its implications for HCC treatment strategies.

This study systematically uncovered the pivotal role of PAQR5 in HCC through a combination of functional enrichment and phenotypic analyses, supported by in vitro and ex vivo experimental validation. KEGG enrichment analysis indicated that high PAQR5 expression is associated with the endomembrane system, biomass regulation, and various metabolic processes, including lipid, small molecule, and organic acid metabolism. These observations suggest that PAQR5 may influence cancer cell metabolism and maintain endoplasmic reticulum integrity, both of which are essential for cellular homeostasis. The association with the endoplasmic reticulum implies that PAQR5 may also be involved in protein folding and stress response mechanisms, which are frequently dysregulated in cancer. Furthermore, GSEA analysis showed that elevated PAQR5 expression is linked to the activation of key oncogenic pathways, such as EMT, the G2M checkpoint, and E2F target genes. These pathways play pivotal roles in tumor progression. EMT, a process where cells undergo a transition from an epithelial to a mesenchymal state, drives metastasis by reducing cell adhesion and enhancing motility, thereby facilitating cell dissemination in HCC [80]. The G2M checkpoint is essential for genomic stability, ensuring DNA integrity before cells enter mitosis. In cancer, dysregulation of the cell cycle, including G2M checkpoint disruption, allows cells with DNA damage to bypass the checkpoint and proceed to mitosis, promoting tumor development [81–83]. The upregulation of angiogenic and inflammatory response pathways further substantiates the role of PAQR5 in fostering a tumorigenic microenvironment in HCC. GSVA revealed that PAQR5 expression significantly correlates with various phenotypes, including angiogenesis, apoptosis, cell cycle regulation, EMT, and metastasis. The strong association with metastatic and invasive traits suggests that PAQR5 may be crucial in enhancing the metastatic potential of HCC cells. Its correlation with apoptosis and DNA damage/repair processes implies that PAQR5 could affect cell survival and genomic stability, thereby promoting tumor progression. The pronounced correlation between PAQR5 and EMT, hypoxia, and stemness phenotypes suggests its involvement in increasing the invasiveness and drug resistance of HCC cells. The NF-κB signaling pathway, commonly dysregulated in cancer, plays a key role in promoting malignancy, accelerating cancer progression, and inducing resistance to radiotherapy [84]. Evidence shows that mPR proteins can activate the intracellular NF-κB signaling pathway, thereby influencing cellular functions [17]. Experimental data demonstrated that PAQR5 promotes HCC cell proliferation, invasion, and EMT by activating the NF-κB signaling pathway, with regulatory effects on p65 phosphorylation and NF-κB activation mediated *via* the ERK pathway. These results highlight PAQR5 as a promising therapeutic target for HCC, especially in the context of strategies aimed at curbing tumor growth, preventing metastasis, and overcoming resistance to conventional treatments.

This study presents several significant findings. The differential expression of PAQR5 in tumors was analyzed across multiple comprehensive datasets and validated at the spatial transcriptome level, as well as through qRT-PCR and western blotting, thereby enhancing the robustness of the results. Additionally, the potential mechanism by which PAQR5 mediates the NF-κB pathway in HCC was elucidated and corroborated through in vitro and in vivo experiments, confirming the associated phenotypic effects.

However, some limitations must be acknowledged. First, the datasets used lacked detailed information on chemotherapy and targeted therapy, limiting the ability to assess patient prognosis comprehensively., Secondly, additional studies are needed to clarify the specific mechanisms by which PAQR5 regulates the NF-κB signaling pathway in HCC.

In investigating the relationship between PAQR5 expression, immune infiltration, functional enrichment, and tumor phenotype in HCC, this study employed multiple levels of validation, including spatial transcriptomics and ex vivo experiments. The results demonstrated that PAQR5 activates the NF-κB signaling pathway by inducing ERK phosphorylation, thereby promoting the proliferation, invasion, and EMT of HCC cells. These findings suggest that PAQR5 is not only a promising independent prognostic factor but also closely linked to immune infiltration and cancer progression in HCC. This study provides a comprehensive understanding of PAQR5’s role in HCC and supports its potential as a novel prognostic biomarker.

## Electronic supplementary material

Below is the link to the electronic supplementary material.

Supplementary Material 1

Supplementary Material 2

Supplementary Material 3

Supplementary Material 4: Fig.S1 Clinicopathologic analysis of PAQR5 and external validation of survival analysis. A PAQR5 transcript expression across 50 tissues is summarized. B Association between PAQR5 expression and tumor G-staging. C Association between PAQR5 expression and tumor clinical stage. D Differences in overall survival between high and low PAQR5 expression groups in the external cohort GSE54236. E Clinicopathological data from the TCGA-LIHC cohort are summarized. *p<0.05; **p<0.01; ***p<0.001.

Supplementary Material 5: Fig.S2 Landscape of key immune molecules and immune cells for PAQR5. A Landscape of key immune molecules for PAQR5. B Landscape of immune cells for PAQR5. *p<0.05; **p<0.01; ***p<0.001.

Supplementary Material 6: Fig.S3 The expression of WNT7B and SH3GL3 was validated in HCC tissues and matched adjacent non-tumor tissues. A-B. Consistent with bioinformatics analysis, western blot assay validated that WNT7B was notably upregulated in 18 human HCC tissues, while SH3GL3 was greatly decreased in HCC specimens compared with matched adjacent non-malignant tissues. *p<0.05.

Supplementary Material 7: Fig.S4 Further GSEA validation, drug screening and chemotherapy sensitivity analysis. A Further GSEA validation across multiple external HCC cohorts. B Small molecule targeted drug screening. C Chemotherapy drug sensitivity analysis. *p<0.05; **p<0.01; ***p<0.001.

Supplementary Material 8: Fig.S5 Epigenetic and genomic mechanisms of dysregulated PAQR5 expression. A Analysis of methylation levels in the 5' untranslated region (5' UTR) of PAQR5. B RNA editing level analysis between tumor and normal patients. C Genetic causal variation between PAQR5 and HCC. D Association between transcription factor expression levels and peak intensity. E, F The overall types of peak locations. G Identification of five peaks associated with PAQR5 transcription, which may indicate regions involved in the regulation of PAQR5 expression. H Genome copy number variation (CNV) analysis, based on GISTIC scores. I Correlation between PAQR5 copy number scores calculated by Gistic2 and PAQR5 mRNA expression. *p<0.05; **p<0.01; ***p<0.001.

Supplementary Material 9: Fig.S6 The m6A level of 5' UTR-PAQR5 was prominently reduced in HCC, and W-13 or Dasatinib effectively inhibited the expression of PAQR5 in HCC cells at a dose-dependent manner. A-B the m6A RIP-qPCR analysis confirmed that the m6A level of 5' UTR-PAQR5 was prominently decreased in HCC specimens and HCC cell lines: Hep3B and MHCC-97 compared with adjacent non-tumor tissues or MIHA respectively. C-D W-13 or Dasatinib effectively mitigated the expression of PAQR5 in Hep3B and MHCC-97H cells at a dose-dependent manner. *p<0.05.

Supplementary Material 10: Fig.S7 PAQR5 promotes HCC cell viability via the activation of NF- B signaling. A. Depletion of PAQR5 in Hep3B and MHCC-97H cells inhibited the HCC cell viability. B. Overexpression of PAQR5 in Hep3B and MHCC-97H cells greatly increased the cell viability, while targeting NF- B signaling partly abrogated the overexpression of PAQR5 induced the increase the cell viability. *p<0.05.

Supplementary Material 11: Fig.S8 PAQR5 expression in HCC cell lines compared to the immortalized normal hepatic cell line MIHA. *p<0.05; **p<0.01; ***p<0.001.

Supplementary Material 12: Fig.S9 PAQR5 facilitated the expression of PD-L1 through the activation of ERK/NF- B signaling. A. the expression of PAQR5 in mouse HCC cell line: Hepa 1-6 after infection of shRNA#1, shRNA#2 and shRNA#3 lentivirus. B-D. Knockdown of PAQR5 in Hepa 1-6, Hep3B and MHCC-97H cells significantly decreased the expression level of PD-L1. E-F. Over-expression of PAQR5 in HCC cells significantly elevated the PD-L1 level, whereas targeting ERK signaling by ERK inhibitor U0126 notably abrogated the over-expression of PAQR5 induced the increase of PD-L1 expression. G-H. Over-expression of PAQR5 greatly facilitated the expression of PD-L1 in HCC cells, while inhibiting the phosphorylation of p65 could notably reverse the overexpression of PAQR5 induced the expression of PD-L1. *p<0.05.

Supplementary Material 13: Fig.S10 Overexpression of PAQR5 in HCC cells repressed the anticancer immunity. A. the HCC cells expressing PAQR5 were co-cultured for 72h with or without activated peripheral blood mononuclear cells (PBMCs) as described in the Methods section, then the HCC cell viability was detected by using MTT assay. B. HCC cells intervened as described in panel (A) were collected to examine the cell apoptosis by flow cytometer. C. qRT-PCR was conducted to examine the expression level of perforin-1, granzyme and granulysin in PBMCs co-cultured with HCC cells expressing PAQR5. D-E. Soluble INF- and IL-2 levels in the supernatants of co-cultures containing HCC cells expressing PAQR5 and PBMCs as detected by Elisa assay. *p<0.05

Supplementary Material 14: Fig.S11 Targeting PAQR5 in HCC cells enhanced the anticancer immunity. A. the HCC cells of PAQR5 depletion were co-cultured for 72h with or without activated peripheral blood mononuclear cells (PBMCs) as described in the Methods section, then the HCC cell viability was detected by using MTT assay. B. HCC cells treated as described in panel (A) were collected to examine the cell apoptosis by flow cytometer. C. qRT-PCR was performed to test the expression level of perforin-1, granzyme and granulysin in PBMCs co-cultured with HCC cells with PAQR5 knockdown. D-E. Soluble INF- and IL-2 levels in the supernatants of co-cultures containing HCC cells with PAQR5 knockdown and PBMCs as detected by Elisa assay. *p<0.05.

## Data Availability

No datasets were generated or analysed during the current study.

## Abbreviations

PAQR5: Progesterone and Adipose Q Receptor 5
HCC: Hepatocellular Carcinoma
EMT: Epithelial-Mesenchymal Transition
Sc-RNA: Single-cell RNA
ST: Spatial Transcriptome
IHC: Immunohistochemical
mPR: membrane Progesterone Receptors
DEG: Differently Expressed Genes
HPA: Human Protein Atlas
ROC: Receiver Operating Characteristic
AUC: Area Under the Curve
HR: Hazard Ratios
OS: Overall Survival
PFI: Progression Free Interval
DSS: Disease-Specific Survival
GSVA: Gene Set Variation Analysis
GO: Gene Ontology
KEGG: Kyoto Encyclopedia of Genes and Genomes
GSEA: Gene Set Enrichment Analysis
PCA: Principal Component Analysis
UMAP: Uniform Manifold Approximation and Projection
TME: Tumor Microenvironment
ATAC: Assay for Transposase-Accessible Chromatin
CTRP: Cancer Therapeutics Response Portal
GTEx: Genotype-Tissue Expression
5’ UTR: 5’ Untranslated Region;
MHC: Major Histocompatibility Complex
CNV: Copy Number Variation
PPI: Protein-Protein Interaction
