# Supplementary figures and images for: PAQR5 drives the malignant progression and shapes the immunosuppressive microenvironment of hepatocellular carcinoma by activating the NF-κB signaling
Source: Biomark Res. 2025 May 7;13:70. doi: 10.1186/s40364-025-00785-z (PMC12060467; doi:10.1186/s40364-025-00785-z)

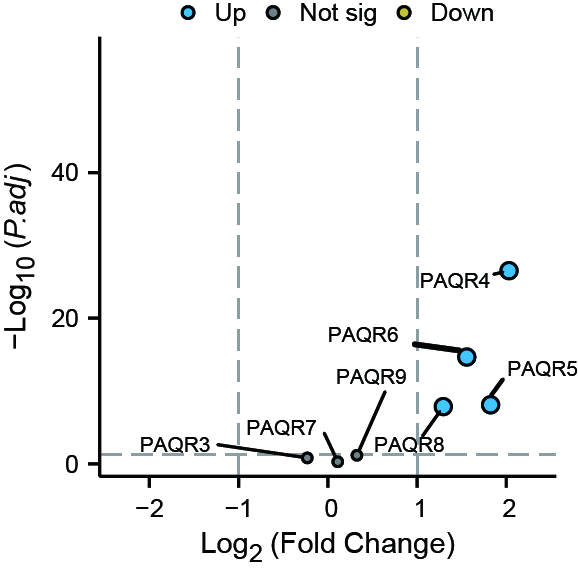

Supplement: Supplementary file 2 — Supplementary Material 2 [file 40364_2025_785_MOESM2_ESM.tif]

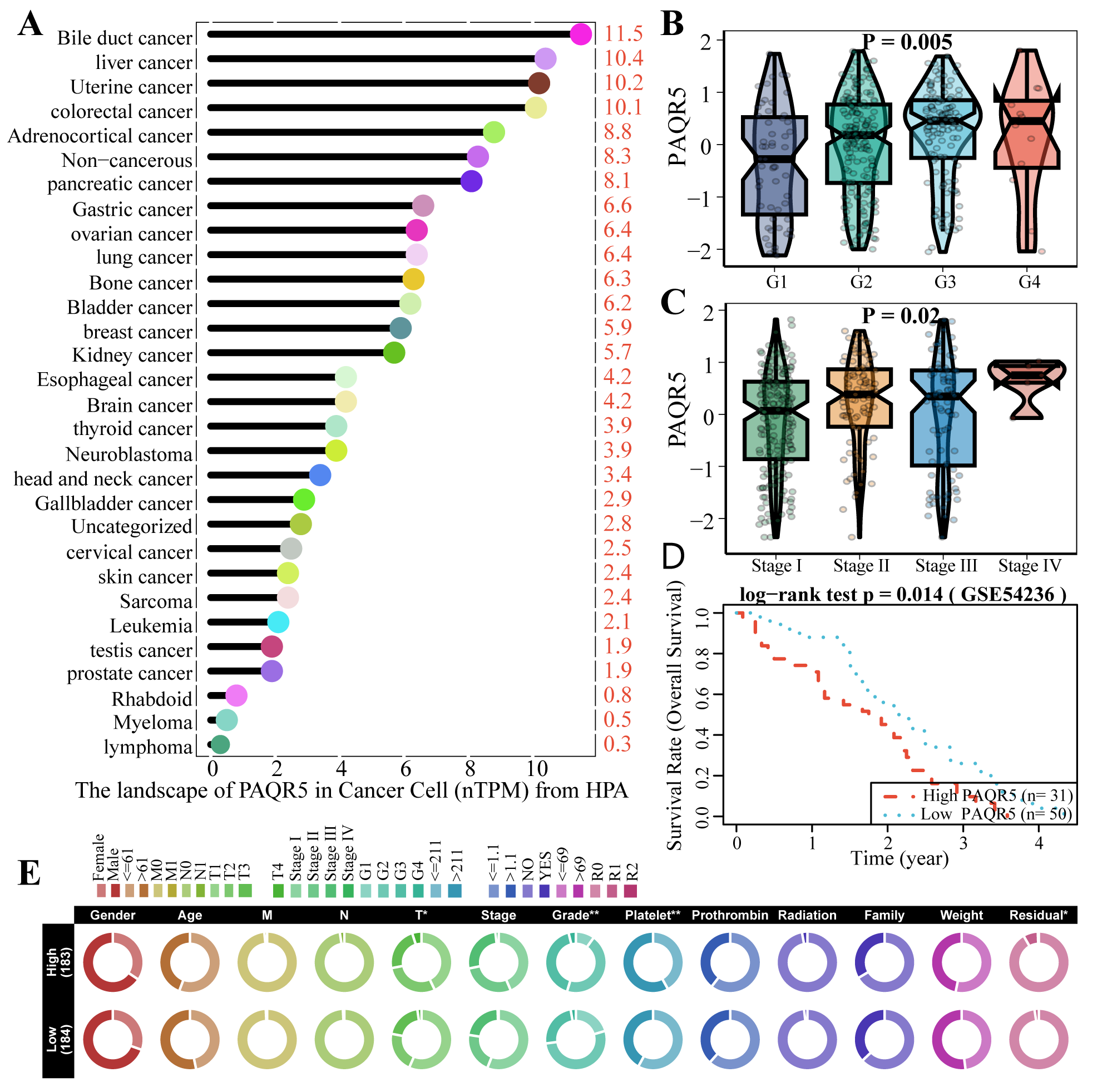

Supplement: Supplementary file 4 — Supplementary Material 4: Fig.S1 Clinicopathologic analysis of PAQR5 and external validation of survival analysis. A PAQR5 transcript expression across 50 tissues is summarized. B Association between PAQR5 expression and tumor G-staging. C Association between PAQR5 expression and tumor clinical stage. D Differences in overall survival between high and low PAQR5 expression groups in the external cohort GSE54236. E Clinicopathological data from the TCGA-LIHC cohort are summarized. *p<0.05; **p<0.01; ***p<0.001. [file 40364_2025_785_MOESM4_ESM.tif]

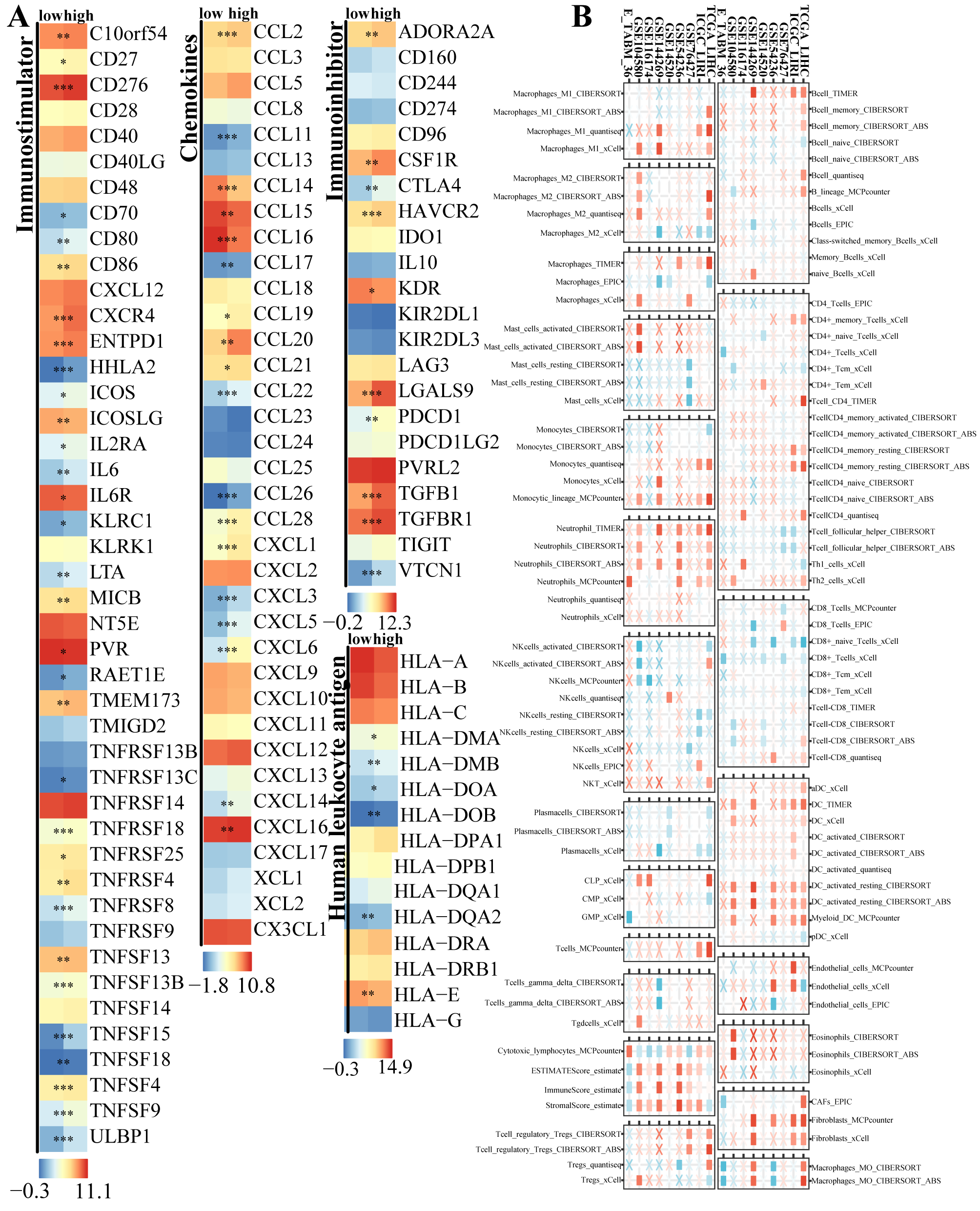

Supplement: Supplementary file 5 — Supplementary Material 5: Fig.S2 Landscape of key immune molecules and immune cells for PAQR5. A Landscape of key immune molecules for PAQR5. B Landscape of immune cells for PAQR5. *p<0.05; **p<0.01; ***p<0.001. [file 40364_2025_785_MOESM5_ESM.tif]

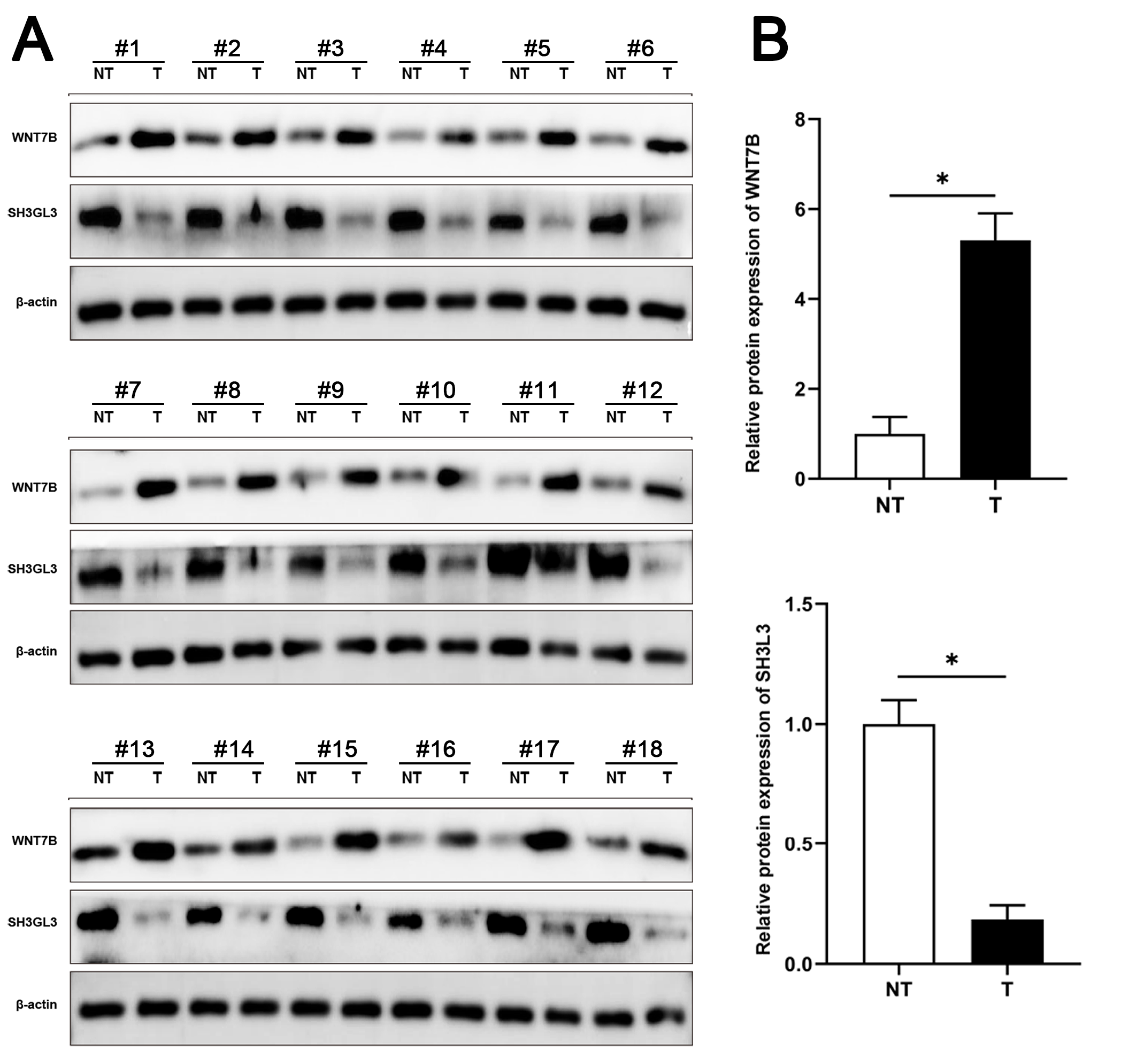

Supplement: Supplementary file 6 — Supplementary Material 6: Fig.S3 The expression of WNT7B and SH3GL3 was validated in HCC tissues and matched adjacent non-tumor tissues. A-B. Consistent with bioinformatics analysis, western blot assay validated that WNT7B was notably upregulated in 18 human HCC tissues, while SH3GL3 was greatly decreased in HCC specimens compared with matched adjacent non-malignant tissues. *p<0.05. [file 40364_2025_785_MOESM6_ESM.tif]

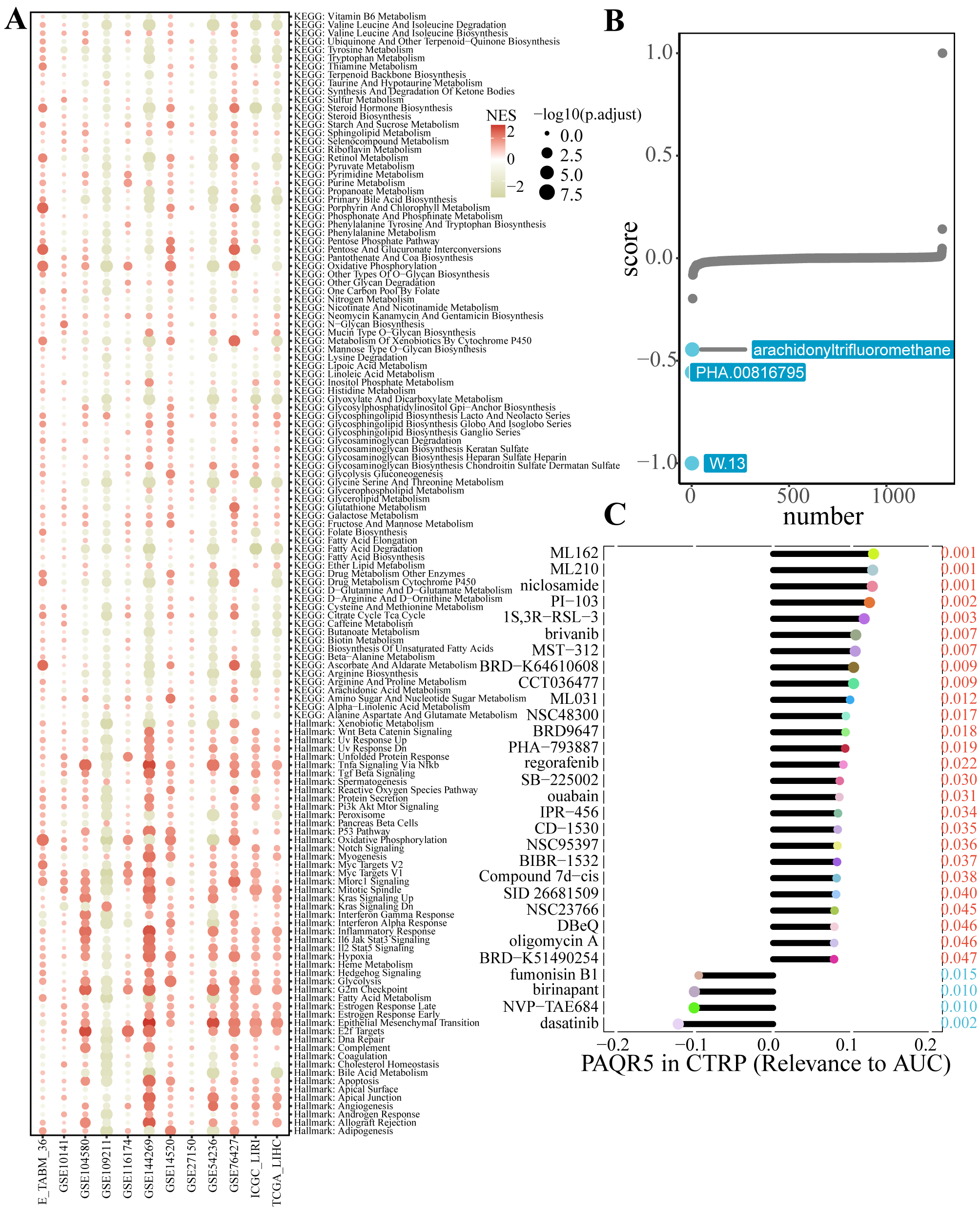

Supplement: Supplementary file 7 — Supplementary Material 7: Fig.S4 Further GSEA validation, drug screening and chemotherapy sensitivity analysis. A Further GSEA validation across multiple external HCC cohorts. B Small molecule targeted drug screening. C Chemotherapy drug sensitivity analysis. *p<0.05; **p<0.01; ***p<0.001. [file 40364_2025_785_MOESM7_ESM.tif]

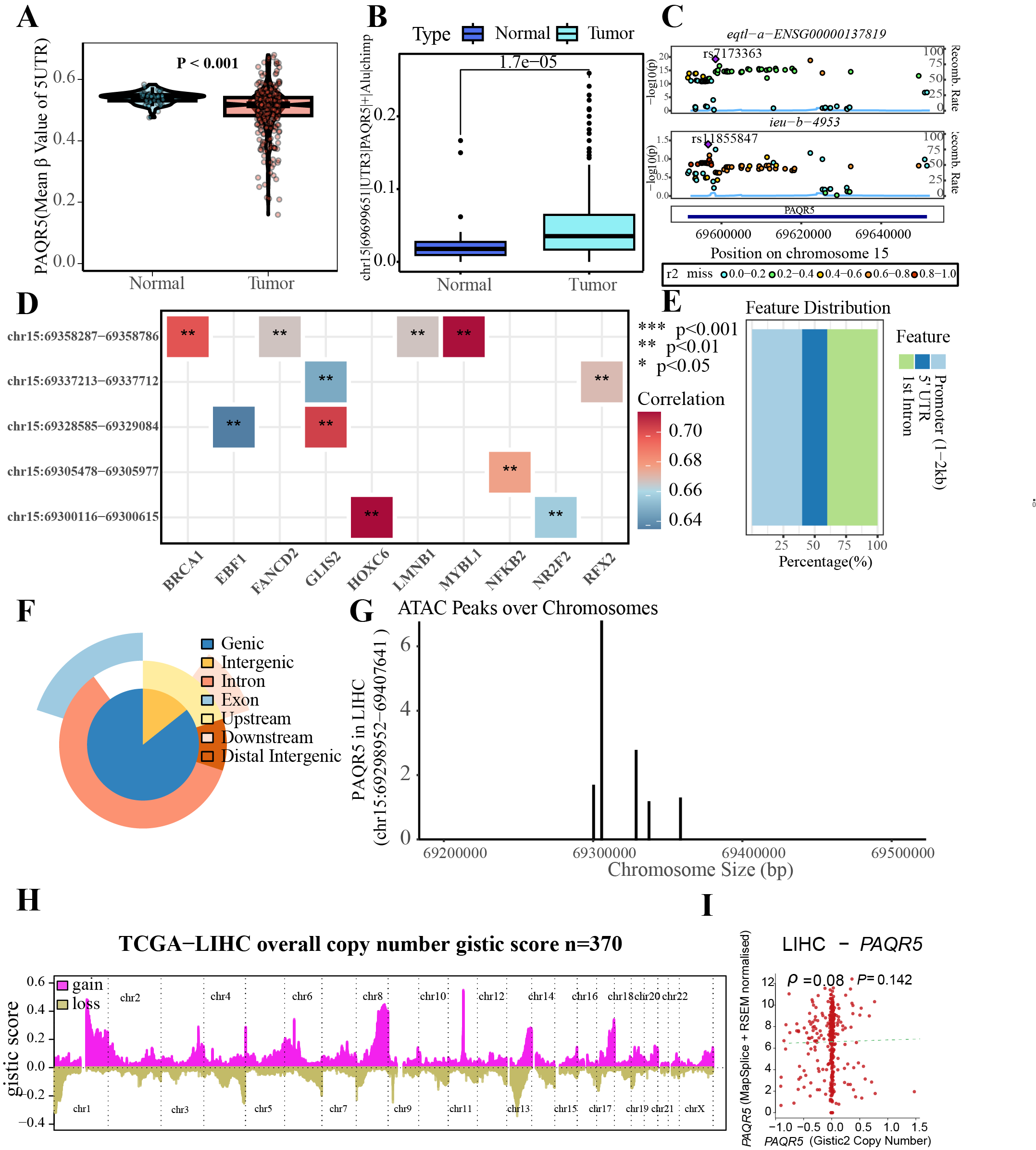

Supplement: Supplementary file 8 — Supplementary Material 8: Fig.S5 Epigenetic and genomic mechanisms of dysregulated PAQR5 expression. A Analysis of methylation levels in the 5' untranslated region (5' UTR) of PAQR5. B RNA editing level analysis between tumor and normal patients. C Genetic causal variation between PAQR5 and HCC. D Association between transcription factor expression levels and peak intensity. E, F The overall types of peak locations. G Identification of five peaks associated with PAQR5 transcription, which may indicate regions involved in the regulation of PAQR5 expression. H Genome copy number variation (CNV) analysis, based on GISTIC scores. I Correlation between PAQR5 copy number scores calculated by Gistic2 and PAQR5 mRNA expression. *p<0.05; **p<0.01; ***p<0.001. [file 40364_2025_785_MOESM8_ESM.tif]

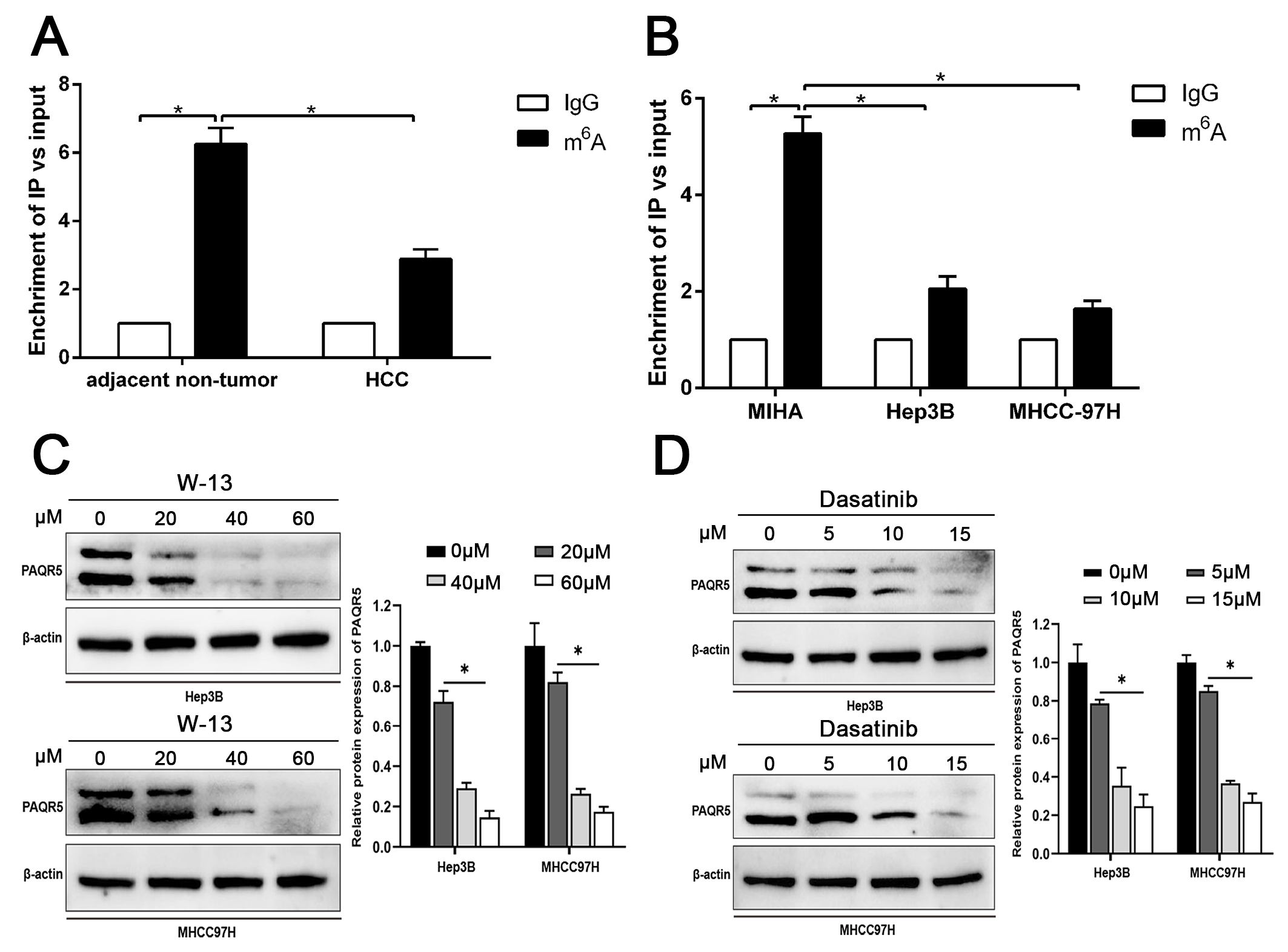

Supplement: Supplementary file 9 — Supplementary Material 9: Fig.S6 The m6A level of 5' UTR-PAQR5 was prominently reduced in HCC, and W-13 or Dasatinib effectively inhibited the expression of PAQR5 in HCC cells at a dose-dependent manner. A-B the m6A RIP-qPCR analysis confirmed that the m6A level of 5' UTR-PAQR5 was prominently decreased in HCC specimens and HCC cell lines: Hep3B and MHCC-97 compared with adjacent non-tumor tissues or MIHA respectively. C-D W-13 or Dasatinib effectively mitigated the expression of PAQR5 in Hep3B and MHCC-97H cells at a dose-dependent manner. *p<0.05. [file 40364_2025_785_MOESM9_ESM.tif]

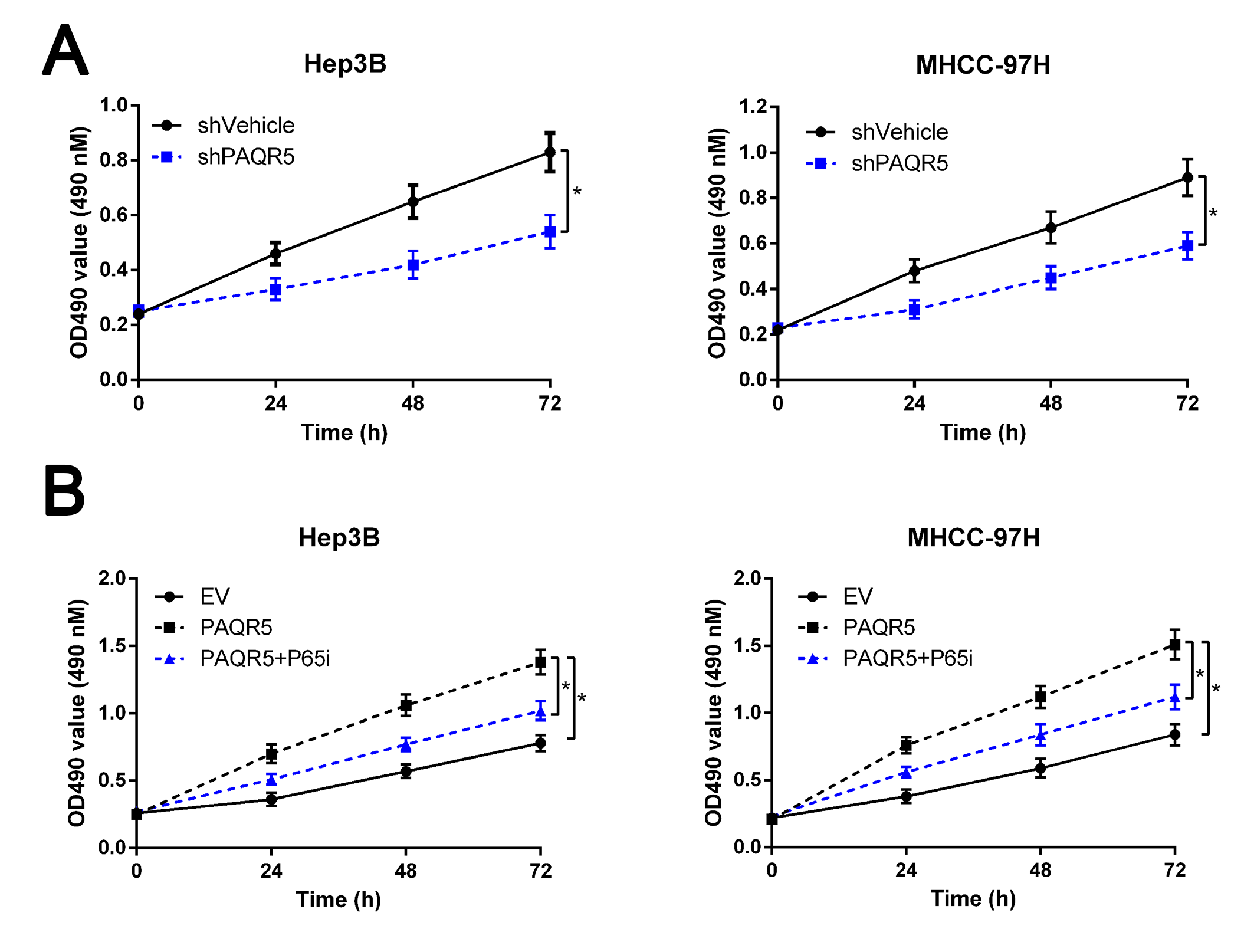

Supplement: Supplementary file 10 — Supplementary Material 10: Fig.S7 PAQR5 promotes HCC cell viability via the activation of NF- B signaling. A. Depletion of PAQR5 in Hep3B and MHCC-97H cells inhibited the HCC cell viability. B. Overexpression of PAQR5 in Hep3B and MHCC-97H cells greatly increased the cell viability, while targeting NF- B signaling partly abrogated the overexpression of PAQR5 induced the increase the cell viability. *p<0.05. [file 40364_2025_785_MOESM10_ESM.tif]

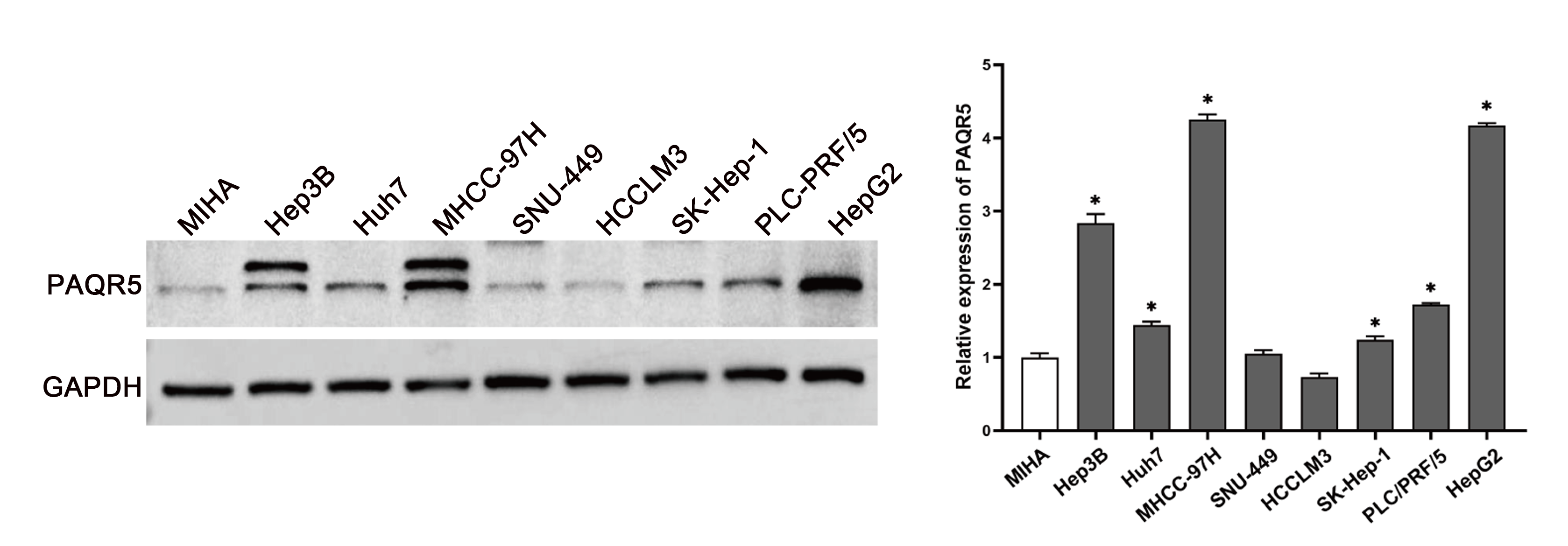

Supplement: Supplementary file 11 — Supplementary Material 11: Fig.S8 PAQR5 expression in HCC cell lines compared to the immortalized normal hepatic cell line MIHA. *p<0.05; **p<0.01; ***p<0.001. [file 40364_2025_785_MOESM11_ESM.tif]

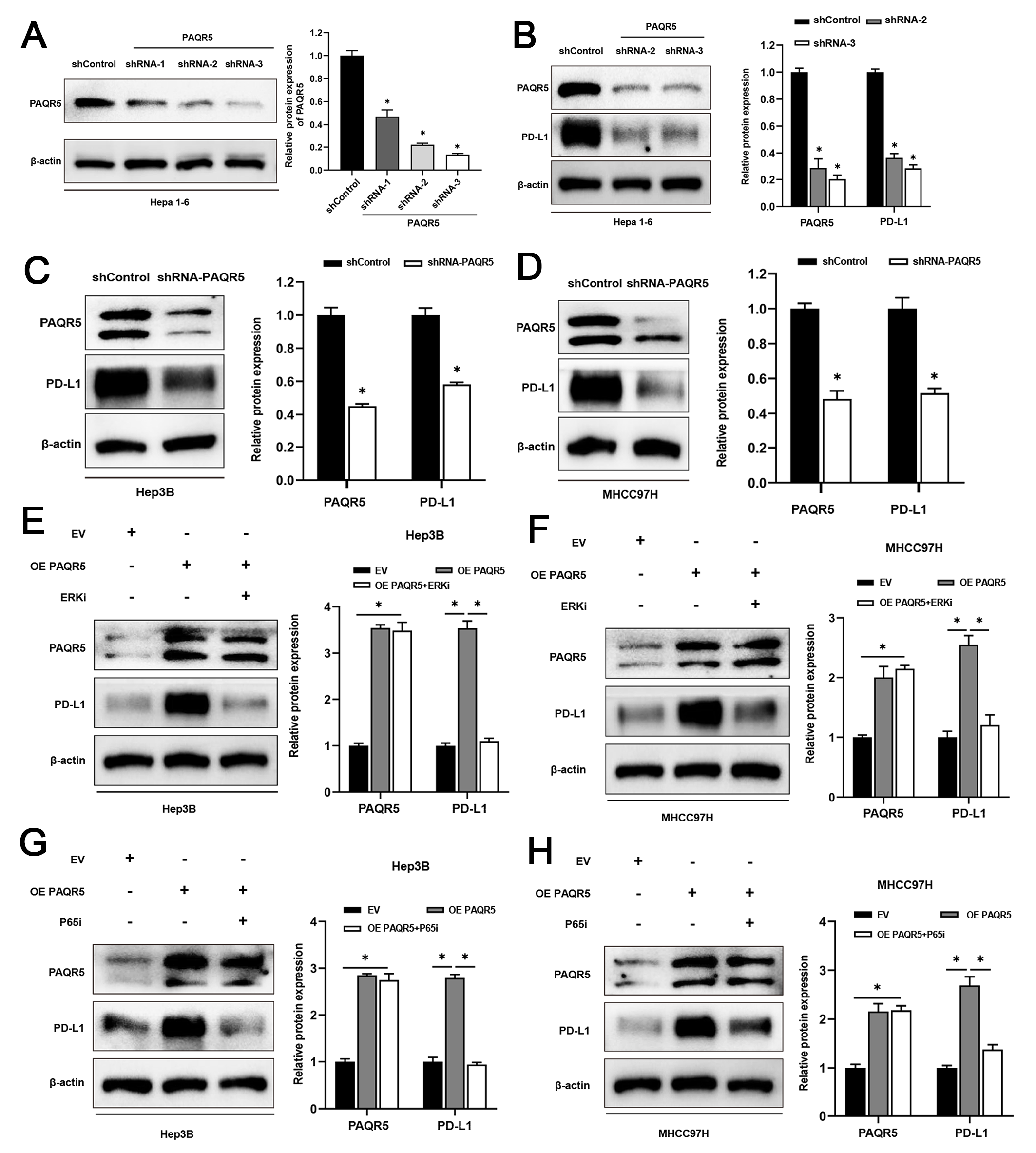

Supplement: Supplementary file 12 — Supplementary Material 12: Fig.S9 PAQR5 facilitated the expression of PD-L1 through the activation of ERK/NF- B signaling. A. the expression of PAQR5 in mouse HCC cell line: Hepa 1-6 after infection of shRNA#1, shRNA#2 and shRNA#3 lentivirus. B-D. Knockdown of PAQR5 in Hepa 1-6, Hep3B and MHCC-97H cells significantly decreased the expression level of PD-L1. E-F. Over-expression of PAQR5 in HCC cells significantly elevated the PD-L1 level, whereas targeting ERK signaling by ERK inhibitor U0126 notably abrogated the over-expression of PAQR5 induced the increase of PD-L1 expression. G-H. Over-expression of PAQR5 greatly facilitated the expression of PD-L1 in HCC cells, while inhibiting the phosphorylation of p65 could notably reverse the overexpression of PAQR5 induced the expression of PD-L1. *p<0.05. [file 40364_2025_785_MOESM12_ESM.tif]

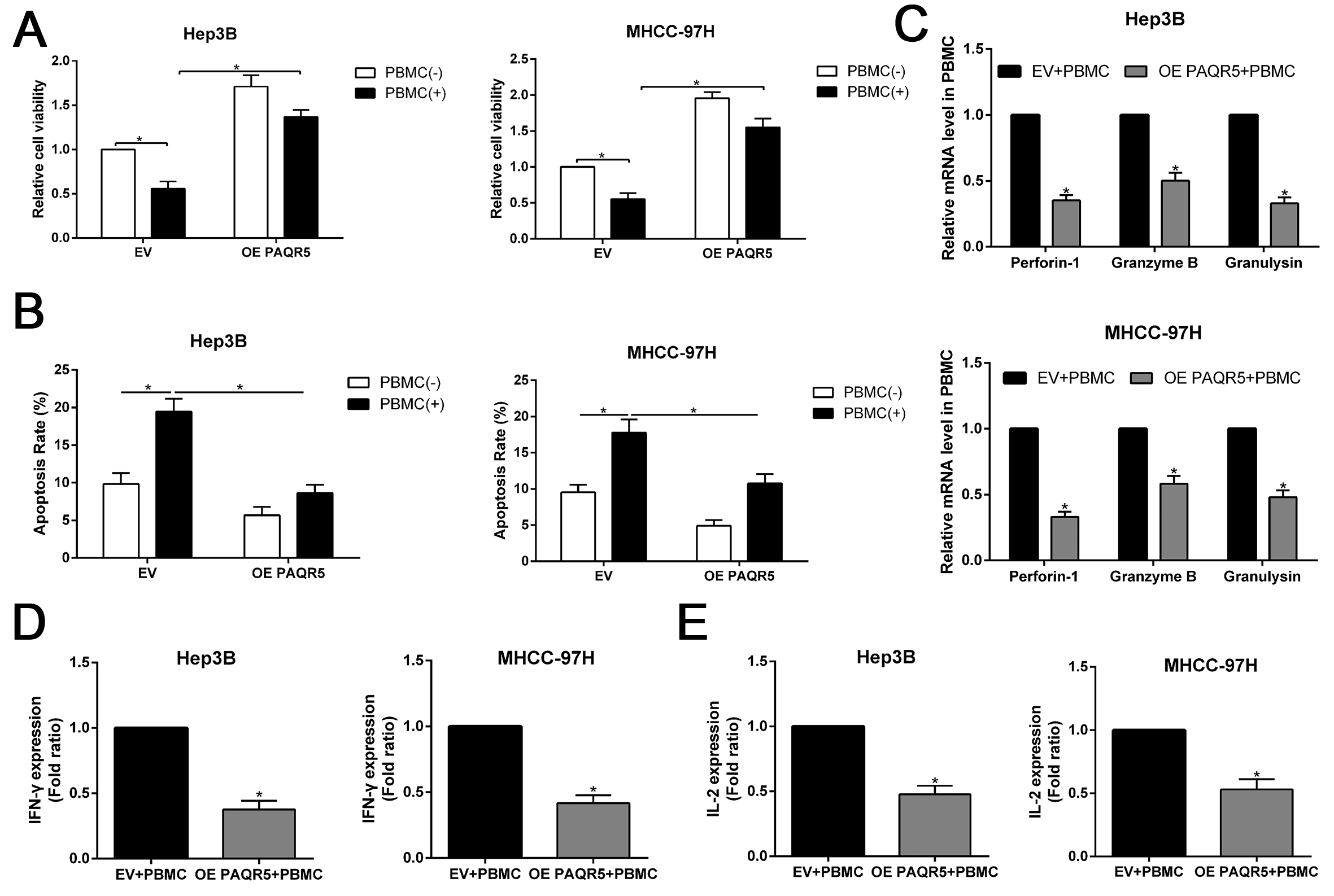

Supplement: Supplementary file 13 — Supplementary Material 13: Fig.S10 Overexpression of PAQR5 in HCC cells repressed the anticancer immunity. A. the HCC cells expressing PAQR5 were co-cultured for 72h with or without activated peripheral blood mononuclear cells (PBMCs) as described in the Methods section, then the HCC cell viability was detected by using MTT assay. B. HCC cells intervened as described in panel (A) were collected to examine the cell apoptosis by flow cytometer. C. qRT-PCR was conducted to examine the expression level of perforin-1, granzyme and granulysin in PBMCs co-cultured with HCC cells expressing PAQR5. D-E. Soluble INF- and IL-2 levels in the supernatants of co-cultures containing HCC cells expressing PAQR5 and PBMCs as detected by Elisa assay. *p<0.05 [file 40364_2025_785_MOESM13_ESM.tif]

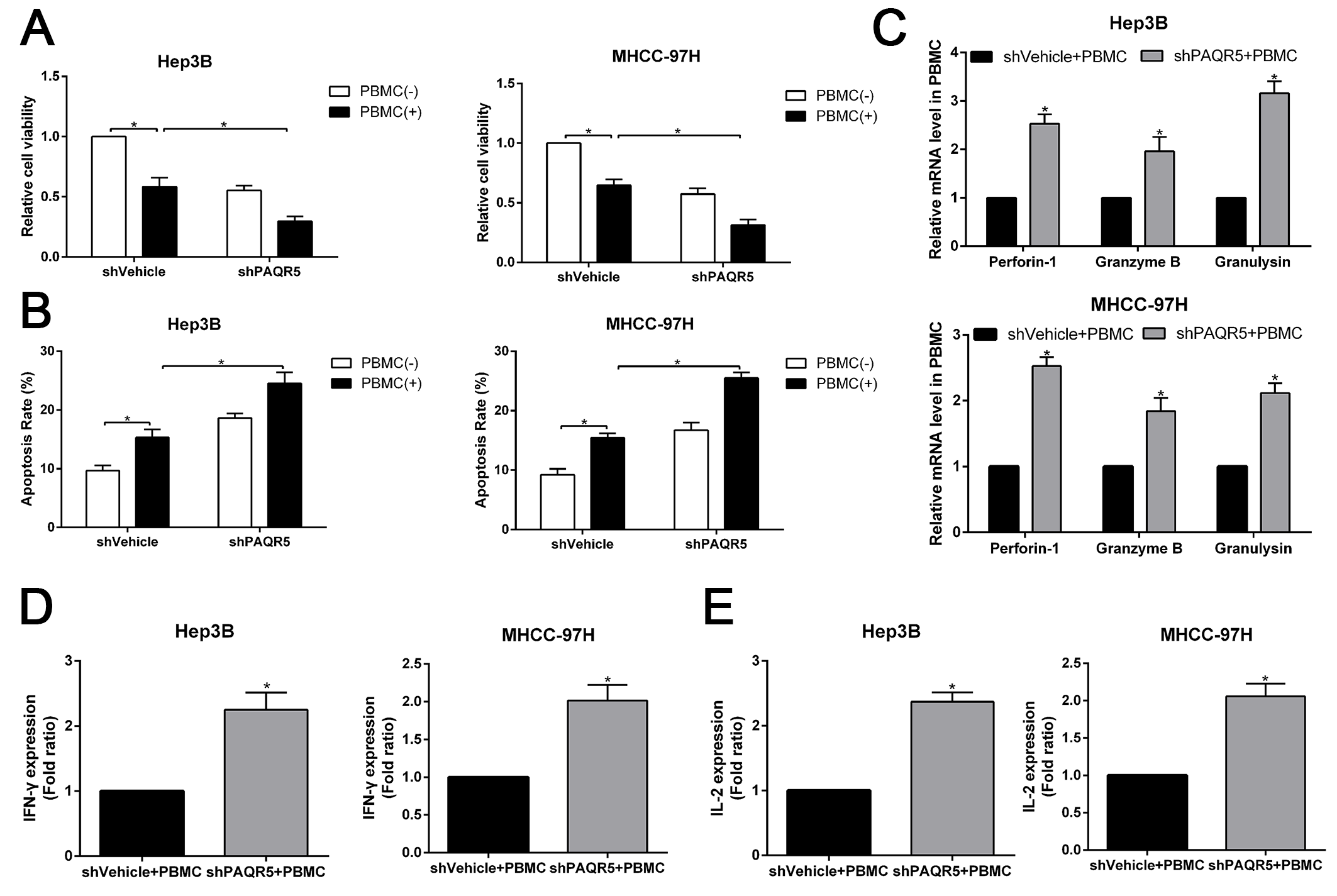

Supplement: Supplementary file 14 — Supplementary Material 14: Fig.S11 Targeting PAQR5 in HCC cells enhanced the anticancer immunity. A. the HCC cells of PAQR5 depletion were co-cultured for 72h with or without activated peripheral blood mononuclear cells (PBMCs) as described in the Methods section, then the HCC cell viability was detected by using MTT assay. B. HCC cells treated as described in panel (A) were collected to examine the cell apoptosis by flow cytometer. C. qRT-PCR was performed to test the expression level of perforin-1, granzyme and granulysin in PBMCs co-cultured with HCC cells with PAQR5 knockdown. D-E. Soluble INF- and IL-2 levels in the supernatants of co-cultures containing HCC cells with PAQR5 knockdown and PBMCs as detected by Elisa assay. *p<0.05. [file 40364_2025_785_MOESM14_ESM.tif]
