# Supplementary material for: PAQR5 drives the malignant progression and shapes the immunosuppressive microenvironment of hepatocellular carcinoma by activating the NF-κB signaling
Source: Biomark Res. 2025 May 7;13:70. doi: 10.1186/s40364-025-00785-z (PMC12060467; doi:10.1186/s40364-025-00785-z)
Supplement: Supplementary file 3 — Supplementary Material 3 [file 40364_2025_785_MOESM3_ESM.docx]

**Supplementary Table 1.** A list of the utilized primary antibodies.

| **Antibody** | **Dilution & Use** | **Company** |
| --- | --- | --- |
| Rabbit anti-PAQR5 | 1:1000 (WB) | Immunoway |
| Rabbit anti-E-cadherin | 1:1000 (WB)  1:100(IHC) | ABclonal |
| Rabbit anti-Vimentin | 1:1000 (WB) 1:100(IHC) | Proteintech |
| Rabbit anti-ERK1/2 | 1:1000 (WB) | Abcam |
| Rabbit anti-p-ERK1/2 (T202/Y204) | 1:1000 (WB) | Cell Signaling Technology |
| Rabbit anti- P65 | 1:1000 (WB) | Cell Signaling Technology |
| Rabbit anti- p-P65 (Ser536) | 1:1000 (WB) | Cell Signaling Technology |
| Rabbit anti- PDL1 | 1:1000 (WB)  1:500(IHC) | Abcam |
| Mouse anti- FOXP3 | 1:400 (IHC) | Proteintech |
| Mouse anti- CD8 | 1:400(IHC) | Proteintech |
| Rabbit anti-ki-67 | 1:500 (IHC) | Abcam |
| Mouse anti-β actin | 1:10000 (WB) | Proteintech |

**Additional file 1: Table S1.** Primers sequences for real-time PCR analysis or m6A RIP-PCR

| **Gene** | **Primer Sequences** |
| --- | --- |
| PAQR5(5′-UTR) | Forward: 5′-GCCTTTAGGTATCTGGACCATTC-3′ |
|  | Reverse: 5′-CTCAGACTCCTCCTCTGTTCTT-3′ |
| Perforin-1 | Forward: 5′-CCTGTGAGGAGAAGAAGAAGAAG-3′ |
|  | Reverse: 5′-TCGTTAATGGAGGTGTGATGG-3′ |
| Granzyme B | Forward: 5′- CTTCCTGATACGAGACGACTTC-3′  Reverse: 5′-CGGCTCCTGTTCTTTGATATTG-3′ |
| Granulysin | Forward:5′-CTGGAAGGGAGAGTGGATTTG-3′ |
|  | Reverse: 5′-GGGCAGGATTTCTCCTCATC-3′ |
| β-actin | Forward: 5′-GGACCTGACTGACTACCTCAT-3′ |
|  | Reverse: 5′-GGACCTGACTGACTACCTCAT-3′ |
